# Supplementary material for: Earliest geometries: A cognitive investigation of Howiesons Poort engraved ostrich eggshells
Source: PLoS One. 2026 Feb 11;21(2):e0338509. doi: 10.1371/journal.pone.0338509 (PMC12893581; doi:10.1371/journal.pone.0338509)
Supplement: S1 Table — (PDF) [file pone.0338509.s001.pdf]

# S1. Dataset EOES

## Diepkloof Rock Shelter

| ID | Tracing | publ. ID | Layer/SU | Square | Nr. Frag | Date (ka BP, Jacobs et al. 2008) | Reference(s) |
|---|---|---|---|---|---|---|---|
| D1 | 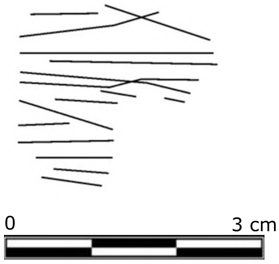  | 2355 | Fred | N7 | 3? | between 65.9 ±3.0 and 61.6 ±2.7 | Texier et al. 2010, fig. 1A |
| D2 | 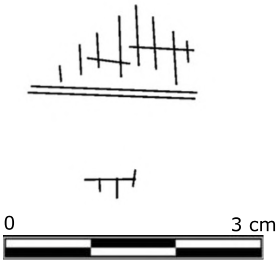  | NA | Frank | N7 | 1 | between 65.9 ±3.0 and 61.6 ±2.7 | Texier et al. 2010, fig. 1B |
| D3 | 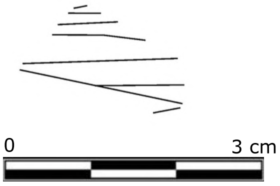  | 2805 | Frank | N7 | 7 | between 65.9 ±3.0 and 61.6 ±2.7 | Texier et al. 2010, fig. 1C |
| D4 | 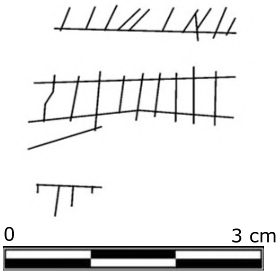  | 2447 / EOES #50 | Frank | N7 | 1 | between 65.9 ±3.0 and 61.6 ±2.7 | Texier et al. 2010, fig. 1D |
| D5 | 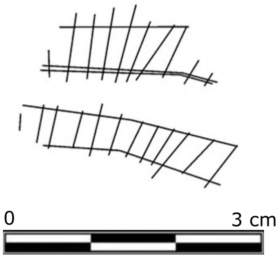  | 2773 | Frank | N7 | 3 | between 65.9 ±3.0 and 61.6 ±2.7 | Texier et al. 2010, fig. 1E |
| D6 | 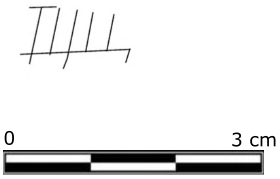  | 2774 | Frank | M7 | 1 | between 65.9 ±3.0 and 61.6 ±2.7 | Texier et al. 2010, fig. 1G |

| | | | | | | | |
|---|---|---|---|---|---|---|---|
| D7 | 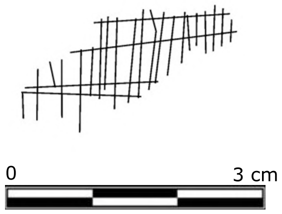 | 3115,<br>3116,<br>3117,<br>3112 | Frank | N7 | 1 | between 65.9 ±3.0 and 61.6 ±2.7 | Texier et al. 2010, fig. 1F |
| D8 | 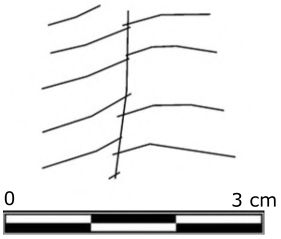 | NA | Frank | N7 | 1 | between 65.9 ±3.0 and 61.6 ±2.7 | Texier et al. 2010, fig. 1H |
| D10 | 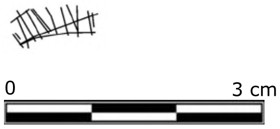 | NA | NA | NA | 1 | ? | Texier et al. 2010, fig. 3A |
| D12 | 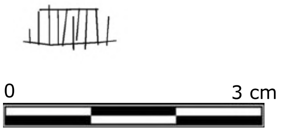 | NA | NA | NA | 1 | ? | Texier et al. 2010, fig. 3D |
| D13 | No tracing. Worn out. Poor quality and not identified as intentionally engraved by the authors. | NA | NA | NA | 3 | ? | Texier et al. 2010, fig. 5A |
| D14 | 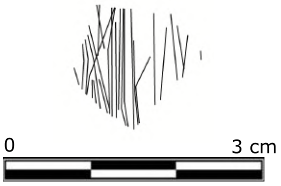 | EOES #256 | Jess | NA | 1 | between 60.8 ±2.6 and 65.1 ±2.8 | Texier et al. 2013, fig. 3a |
| D15 | 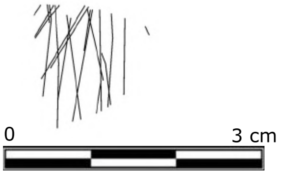 | EOES #255 | Jude | NA | 1 | between 60.8 ±2.6 and 65.1 ±2.8 | Texier et al. 2013, fig. 3b |
| D16 | 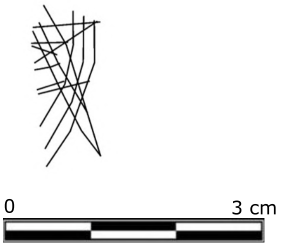 | EOES #414 | Jude | NA | 1 | between 60.8 ±2.6 and 65.1 ±2.8 | Texier et al. 2013, fig. 3c |

| | | | | | | | |
|---|---|---|---|---|---|---|---|
| D17 | 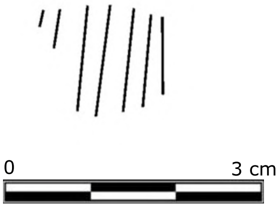  | EOES #368 | Jack | NA | 1 | between 60.8 ±2.6 and 65.1 ±2.8 | Texier et al. 2013, fig. 3d |
| D18 | 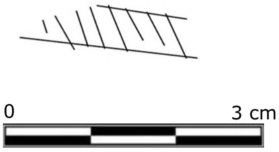  | EOES #5 | Glen | NA | 1 | between 49.1 ±2.2 and 59.2 ±2.7 | Texier et al. 2013, fig. 4a2 |
| D19 | 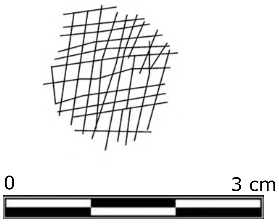  | EOES #47 | Eric | NA | 1 | after 65.9 ±3.0 | Texier et al. 2013, fig. 4b |
| D21 | 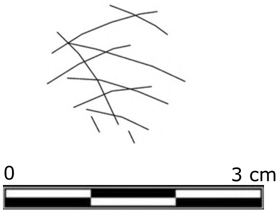  | EOES #95 | Fred | NA | 1 | between 65.9 ±3.0 and 61.6 ±2.7 | Texier et al. 2013, fig. 4d |
| D23 | 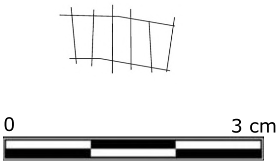  | NA | Frank | from M6-9 to N6-9 | 1 | between 65.9 ±3.0 and 61.6 ±2.7 | Texier et al. 2013, fig. 5,1 |
| D24 | 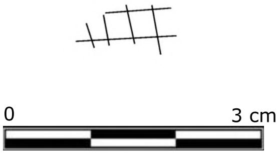  | NA | Frank | from M6-9 to N6-9 | 1 | between 65.9 ±3.0 and 61.6 ±2.7 | Texier et al. 2013, fig. 5,2 |
| D25 | 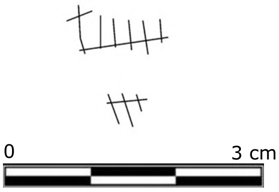  | NA | Frank | from M6-9 to N6-9 | 1 | between 65.9 ±3.0 and 61.6 ±2.7 | Texier et al. 2013, fig. 5,3 |
| D26 | 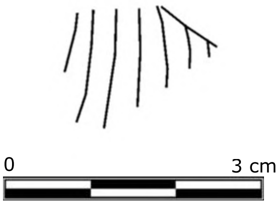  | NA | Frank | from M6-9 to N6-9 | 1 | between 65.9 ±3.0 and 61.6 ±2.7 | Texier et al. 2013, fig. 5,4 |
| D27 | 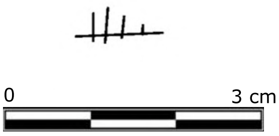  | NA | Frank | from M6-9 to N6-9 | 1 | between 65.9 ±3.0 and 61.6 ±2.7 | Texier et al. 2013, fig. 5,5 |

| | | | | | | | |
|---|---|---|---|---|---|---|---|
| D28 | 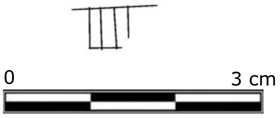 | NA | Frank | from M6-9 to N6-9 | 1 | between 65.9 ±3.0 and 61.6 ±2.7 | Texier et al. 2013, fig. 5,6 |
| D29 | 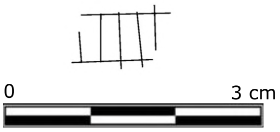 | NA | Frank | from M6-9 to N6-9 | 1 | between 65.9 ±3.0 and 61.6 ±2.7 | Texier et al. 2013, fig. 5,7 |
| D30 | 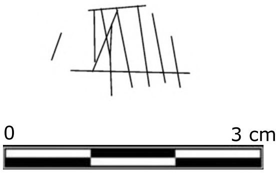 | NA | Frank | from M6-9 to N6-9 | 1 | between 65.9 ±3.0 and 61.6 ±2.7 | Texier et al. 2013, fig. 5,8 |
| D31 | 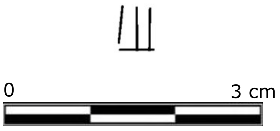 | NA | Frank | from M6-9 to N6-9 | 1 | between 65.9 ±3.0 and 61.6 ±2.7 | Texier et al. 2013, fig. 5,9 |
| D32 | 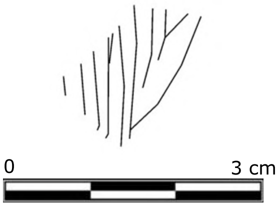 | NA | Frank | from M6-9 to N6-9 | 1 | between 65.9 ±3.0 and 61.6 ±2.7 | Texier et al. 2013, fig. 5,10 |
| D33 | 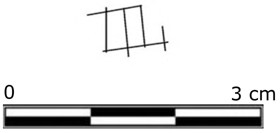 | NA | Frank | from M6-9 to N6-9 | 1 | between 65.9 ±3.0 and 61.6 ±2.7 | Texier et al. 2013, fig. 5,11 |
| D34 | 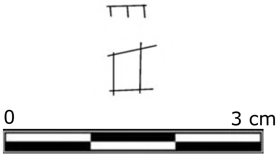 | NA | Frank | from M6-9 to N6-9 | 1 | between 65.9 ±3.0 and 61.6 ±2.7 | Texier et al. 2013, fig. 5,12 |
| D35 | 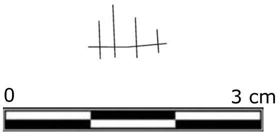 | NA | Frank | from M6-9 to N6-9 | 1 | between 65.9 ±3.0 and 61.6 ±2.7 | Texier et al. 2013, fig. 5,13 |
| D36 | 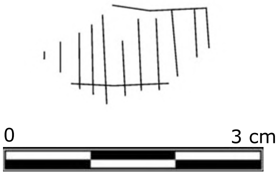 | NA | Frank | from M6-9 to N6-9 | 1 | between 65.9 ±3.0 and 61.6 ±2.7 | Texier et al. 2013, fig. 5,14 |
| D37 | 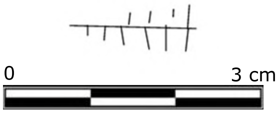 | NA | Frank | from M6-9 to N6-9 | 1 | between 65.9 ±3.0 and 61.6 ±2.7 | Texier et al. 2013, fig. 5,15 |
| D38 | 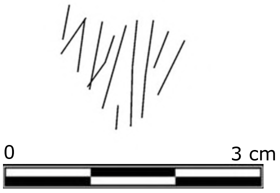 | NA | Frank | from M6-9 to N6-9 | 1 | between 65.9 ±3.0 and 61.6 ±2.7 | Texier et al. 2013, fig. 5,16 |

| | | | | | | | |
|---|---|---|---|---|---|---|---|
| D39 | 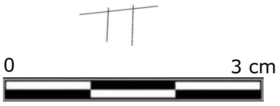 | NA | Frank | from M6-9 to N6-9 | 1 | between 65.9 ±3.0 and 61.6 ±2.7 | Texier et al. 2013, fig. 5,17 |
| D40 | 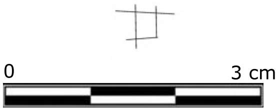 | NA | Frank | from M6-9 to N6-9 | 1 | between 65.9 ±3.0 and 61.6 ±2.7 | Texier et al. 2013, fig. 5,18 |
| D41 | 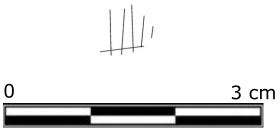 | NA | Frank | from M6-9 to N6-9 | 1 | between 65.9 ±3.0 and 61.6 ±2.7 | Texier et al. 2013, fig. 5,19 |
| D42 | 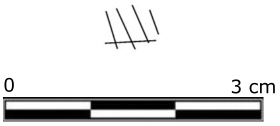 | NA | Frank | from M6-9 to N6-9 | 1 | between 65.9 ±3.0 and 61.6 ±2.7 | Texier et al. 2013, fig. 5,20 |
| D43 | 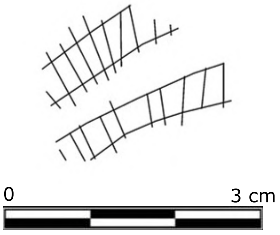 | NA | Frank | from M6-9 to N6-9 | 3 | between 65.9 ±3.0 and 61.6 ±2.7 | Texier et al. 2013, fig. 5,21 |
| D44 | 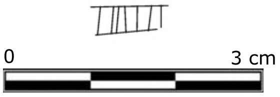 | NA | Frank | from M6-9 to N6-9 | 1 | between 65.9 ±3.0 and 61.6 ±2.7 | Texier et al. 2013, fig. 5,22 |
| D45 | 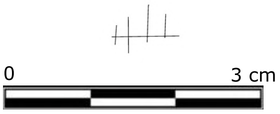 | NA | Frank | from M6-9 to N6-9 | 1 | between 65.9 ±3.0 and 61.6 ±2.7 | Texier et al. 2013, fig. 5,23 |
| D46 | 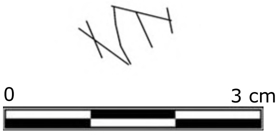 | NA | Frank | from M6-9 to N6-9 | 1 | between 65.9 ±3.0 and 61.6 ±2.7 | Texier et al. 2013, fig. 5,24 |
| D47 | 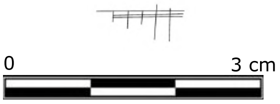 | NA | Frank | from M6-9 to N6-9 | 1 | between 65.9 ±3.0 and 61.6 ±2.7 | Texier et al. 2013, fig. 5,25 |
| D48 | 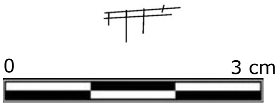 | NA | Frank | from M6-9 to N6-9 | 1 | between 65.9 ±3.0 and 61.6 ±2.7 | Texier et al. 2013, fig. 5,26 |

| | | | | | | | |
|---|---|---|---|---|---|---|---|
| D49 | 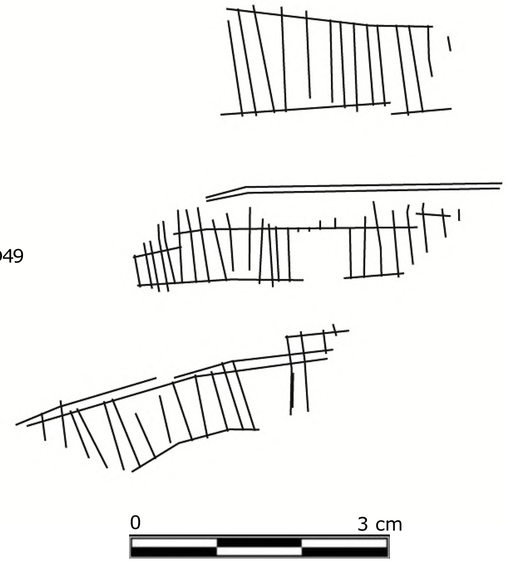 | EOES #6,26, 37, 49, 103, 123, 171, 237, 239, 240 | Frank | N7-8 | 11 | between 65.9 ±3.0 and 61.6 ±2.7 | Texier et al. 2013, fig. 6a, block 1 |
| D50 | 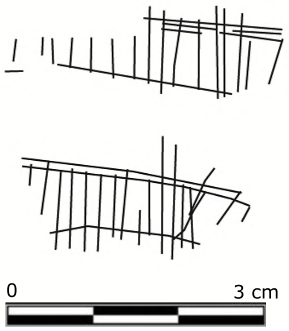 | EOES #126, 159, 235, 238, 242, 242bis | Frank | N7-8 | 6 | between 65.9 ±3.0 and 61.6 ±2.7 | Texier et al. 2013, fig. 6a, block 2 |
| D51 | 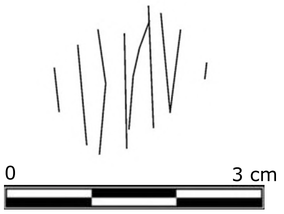 | NA | Danny-Debbi | NA | 1 | after 65.9 ±3.0 | Texier et al. 2013, fig. 7, 27 |
| D52 | 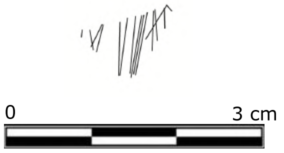 | NA | Danny-Debbi | NA | 1 | after 65.9 ±3.0 | Texier et al. 2013, fig. 7, 28 |
| D53 | 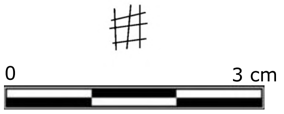 | NA | Edgar | NA | 1 | 65.9 ±3.0 | Texier et al. 2013, fig. 7, 29 |
| D54 | 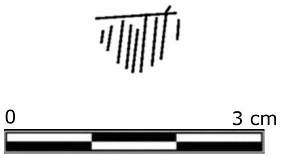 | NA | Edgar | NA | 1 | 65.9 ±3.0 | Texier et al. 2013, fig. 7, 30 |
| D55 | 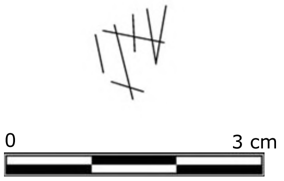 | NA | Edgar | NA | 1 | 65.9 ±3.0 | Texier et al. 2013, fig. 7, 31 |
| D56 | 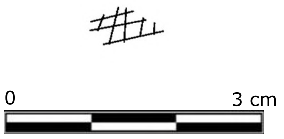 | NA | Ester | NA | 1 | after 65.9 ±3.0 | Texier et al. 2013, fig. 7, 32 |

| | | | | | | | |
|---|---|---|---|---|---|---|---|
| D57 | 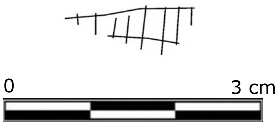 | NA | Ester | NA | 1 | after 65.9 ±3.0 | Texier et al. 2013, fig. 7, 33 |
| D58 | 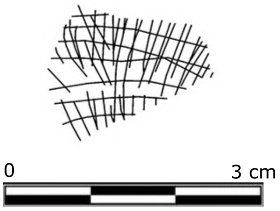 | NA | Ester | NA | 1 | after 65.9 ±3.0 | Texier et al. 2013, fig. 7, 34 |
| D59 | 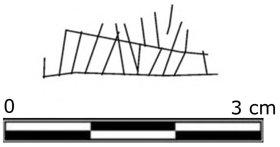 | NA | Eve | NA | 2 | between 65.9 ±3.0 and 61.6 ±2.7 | Texier et al. 2013, fig. 7, 35 |
| D60 | 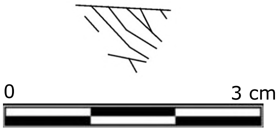 | NA | Eve | NA | 1 | between 65.9 ±3.0 and 61.6 ±2.7 | Texier et al. 2013, fig. 7, 36 |
| D61 | 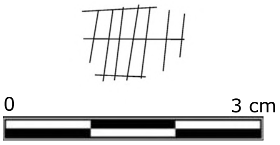 | NA | Eve | NA | 1 | between 65.9 ±3.0 and 61.6 ±2.7 | Texier et al. 2013, fig. 7, 37 |
| D62 | 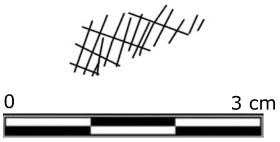 | NA | Eve | NA | 1 | between 65.9 ±3.0 and 61.6 ±2.7 | Texier et al. 2013, fig. 7, 38 |
| D63 | 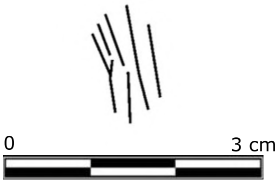 | NA | Eve | NA | 1 | between 65.9 ±3.0 and 61.6 ±2.7 | Texier et al. 2013, fig. 7, 39 |
| D64 | 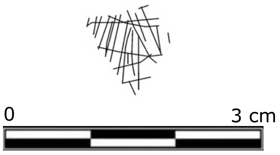 | NA | Eve | NA | 1 | between 65.9 ±3.0 and 61.6 ±2.7 | Texier et al. 2013, fig. 7, 40 |
| D65 | 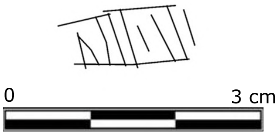 | NA | Eve | NA | 1 | between 65.9 ±3.0 and 61.6 ±2.7 | Texier et al. 2013, fig. 7, 41 |
| D66 | 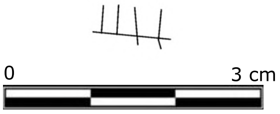 | NA | Fiona | NA | 1 | between 65.9 ±3.0 and 61.6 ±2.7 | Texier et al. 2013, fig. 7, 42 |
| D67 | 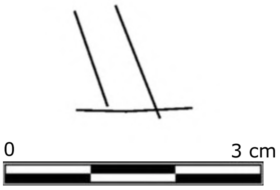 | NA | Fox | NA | 1 | between 65.9 ±3.0 and 61.6 ±2.7 | Texier et al. 2013, fig. 7, 43 |

| | | | | | | | |
|---|---|---|---|---|---|---|---|
| D68 | No tracing. Worn out. Poor quality image | NA | Fox | NA | 1 | between 65.9 ±3.0 and 61.6 ±2.7 | Texier et al. 2013, fig. 7, 44 |
| D69 | 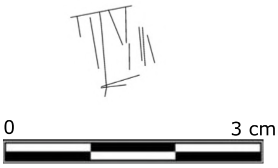  | NA | Fox | NA | 1 | between 65.9 ±3.0 and 61.6 ±2.7 | Texier et al. 2013, fig. 7, 45 |
| D70 | 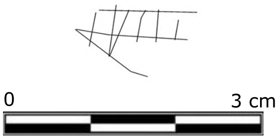  | NA | Fox | NA | 1 | between 65.9 ±3.0 and 61.6 ±2.7 | Texier et al. 2013, fig. 7, 46 |
| D71 | 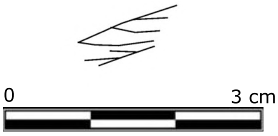  | NA | Fox | NA | 1 | between 65.9 ±3.0 and 61.6 ±2.7 | Texier et al. 2013, fig. 7, 47 |
| D72 | 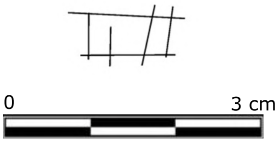  | NA | Fox | NA | 1 | between 65.9 ±3.0 and 61.6 ±2.7 | Texier et al. 2013, fig. 7, 48 |
| D73 | No tracing. Worn out. Poor quality image | NA | Fox | NA | 2 | between 65.9 ±3.0 and 61.6 ±2.7 | Texier et al. 2013, fig. 7, 49 |
| D74 | 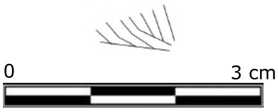  | NA | Frans | NA | 1 | between 65.9 ±3.0 and 61.6 ±2.7 | Texier et al. 2013, fig. 7, 50 |
| D75 | 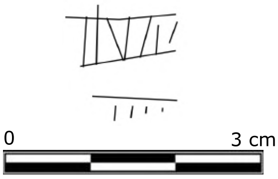  | NA | Frans | NA | 1 | between 65.9 ±3.0 and 61.6 ±2.7 | Texier et al. 2013, fig. 7, 51 |
| D76 | 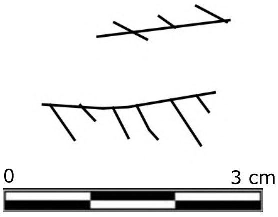  | NA | Frans | NA | 2 | between 65.9 ±3.0 and 61.6 ±2.7 | Texier et al. 2013, fig. 7, 52 |
| D77 | 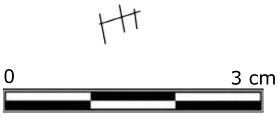  | NA | Frans | NA | 1 | between 65.9 ±3.0 and 61.6 ±2.7 | Texier et al. 2013, fig. 7, 53 |
| D78 | 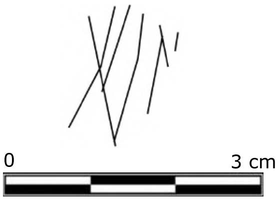  | NA | Frans | NA | 1 | between 65.9 ±3.0 and 61.6 ±2.7 | Texier et al. 2013, fig. 7, 54 |

| | | | | | | | |
|---|---|---|---|---|---|---|---|
| D79 | 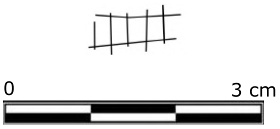 | NA | Frans | NA | 1 | between 65.9 ±3.0 and 61.6 ±2.7 | Texier et al. 2013, fig. 7, 55 |
| D80 | 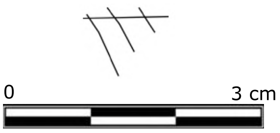 | NA | Frans | NA | 1 | between 65.9 ±3.0 and 61.6 ±2.7 | Texier et al. 2013, fig. 7, 56 |
| D81 | 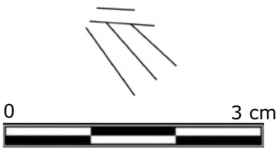 | NA | Frans | NA | 1 | between 65.9 ±3.0 and 61.6 ±2.7 | Texier et al. 2013, fig. 7, 57 |
| D82 | 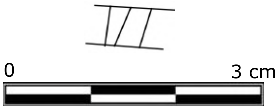 | NA | Fred | NA | 1 | between 65.9 ±3.0 and 61.6 ±2.7 | Texier et al. 2013, fig. 7, 58 |
| D83 | 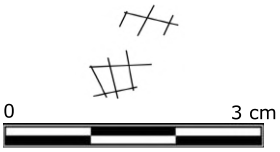 | NA | Fred | NA | 1 | between 65.9 ±3.0 and 61.6 ±2.7 | Texier et al. 2013, fig. 7, 59 |
| D84 | 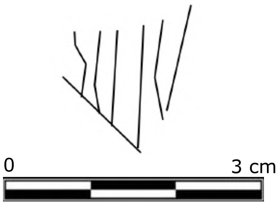 | NA | Fred | NA | 1 | between 65.9 ±3.0 and 61.6 ±2.7 | Texier et al. 2013, fig. 7, 60 |
| D85 | 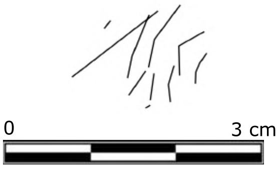 | NA | Fred | NA | 1 | between 65.9 ±3.0 and 61.6 ±2.7 | Texier et al. 2013, fig. 7, 61 |
| D86 | 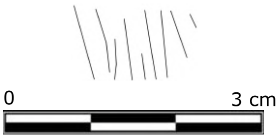 | NA | Fred | NA | 1 | between 65.9 ±3.0 and 61.6 ±2.7 | Texier et al. 2013, fig. 7, 62 |
| D87 | 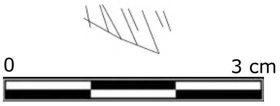 | NA | Fred | NA | 1 | between 65.9 ±3.0 and 61.6 ±2.7 | Texier et al. 2013, fig. 7, 63 |
| D89 | 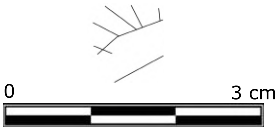 | NA | Fred | NA | 1 | between 65.9 ±3.0 and 61.6 ±2.7 | Texier et al. 2013, fig. 7, 65<br>66 |
| D90 | 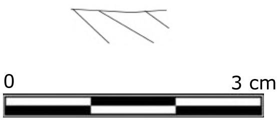 | NA | Fred | NA | 1 | between 65.9 ±3.0 and 61.6 ±2.7 | Texier et al. 2013, fig. 7, |

| | | | | | | | |
|---|---|---|---|---|---|---|---|
| D91 | 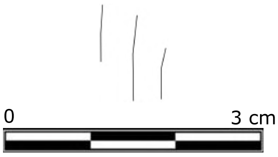 | NA | Fred | NA | 1 | between 65.9 ±3.0 and 61.6 ±2.7 | Texier et al. 2013, fig. 7, 67 |
| D92 | 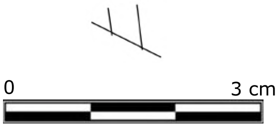 | NA | Fred | NA | 1 | between 65.9 ±3.0 and 61.6 ±2.7 | Texier et al. 2013, fig. 7, 68 |
| D93 | 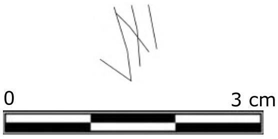 | NA | Fred | NA | 1 | between 65.9 ±3.0 and 61.6 ±2.7 | Texier et al. 2013, fig. 7, 69 |
| D94 | 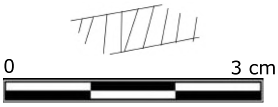 | NA | Fred | NA | 1 | between 65.9 ±3.0 and 61.6 ±2.7 | Texier et al. 2013, fig. 7, 70 |
| D96 | 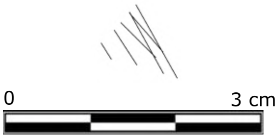 | NA | Fred | NA | 1 | between 65.9 ±3.0 and 61.6 ±2.7 | Texier et al. 2013, fig. 7, 72 |
| D97 | 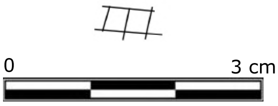 | NA | Fred | NA | 1 | between 65.9 ±3.0 and 61.6 ±2.7 | Texier et al. 2013, fig. 7, 73 |
| D98 | 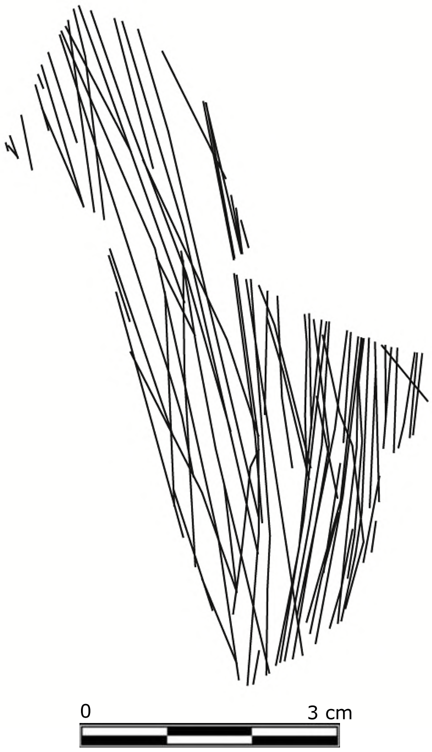 | EOES #58, 59, 60, 61 | Darryl | M9 | 4 | after 65.9 ± 3.0 | Texier et al. 2013, fig. 10 |

# Klipdrift Shelter

| ID | Tracing | publ. ID | Layer/SU | Date (ka BP, Henshilwood et al. 2014) | Reference |
|---|---|---|---|---|---|
| K1 | 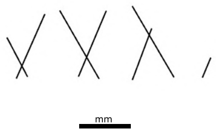 | NA | PAZ | between 59.4 ±4.6 and 60.0 ±4.0 | Henshilwood et al. 2014, fig. 10a |
| K2 | 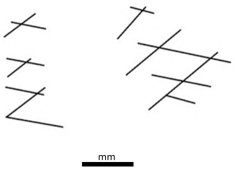 | NA | PAZ | between 59.4 ±4.6 and 60.0 ±4.0 | Henshilwood et al. 2014, fig. 10b |
| K3 | 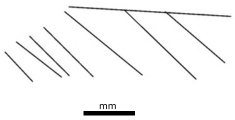 | NA | PBC | 65.5 ± 4.8 | Henshilwood et al. 2014, fig. 10c |
| K4 | 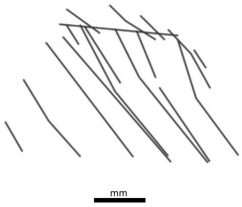 | NA | PBC | 65.5 ± 4.8 | Henshilwood et al. 2014, fig. 10d |
| K5 | 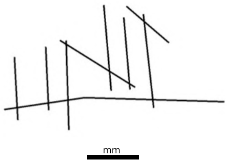 | NA | PPD | 64.6 ± 4.2 | Henshilwood et al. 2014, fig. 10e |
| K6 | 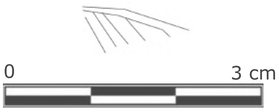 | NA | NA | between 65.5 ±4.8 ka and 59.4 ±4.6 ka | Nel & Haaland 2023, fig. 4 |
| K7 | 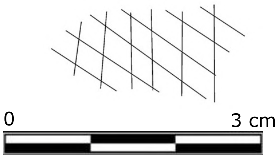 | NA | NA | between 65.5 ±4.8 ka and 59.4 ±4.6 ka | Nel & Haaland 2023, fig. 4 |
| K8 | 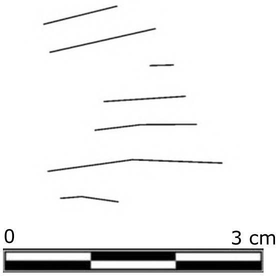 | NA | NA | between 65.5 ±4.8 ka and 59.4 ±4.6 ka | Nel & Haaland 2023, fig. 4 |

| | | | | | |
|---|---|---|---|---|---|
| K9 | 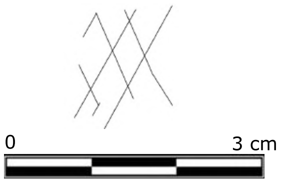 | NA | NA | between 65.5 ±4.8 ka and 59.4 ±4.6 ka | Nel & Haaland 2023, fig. 4 |
| K10 | 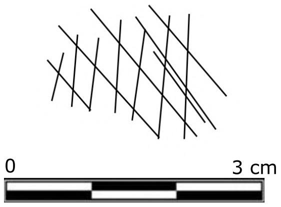 | NA | NA | between 65.5 ±4.8 ka and 59.4 ±4.6 ka | Nel & Haaland 2023, fig. 4 |
| K11 | 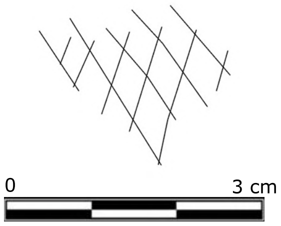 | NA | NA | between 65.5 ±4.8 ka and 59.4 ±4.6 ka | Nel & Haaland 2023, fig. 4 |
| K12 | 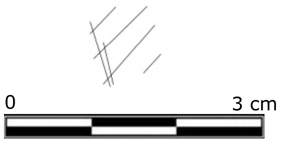 | NA | NA | between 65.5 ±4.8 ka and 59.4 ±4.6 ka | Nel & Haaland 2023, fig. 4 |
| K13 | 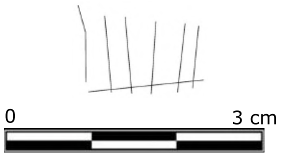 | NA | NA | between 65.5 ±4.8 ka and 59.4 ±4.6 ka | Nel & Haaland 2023, fig. 4 |
| K14 | 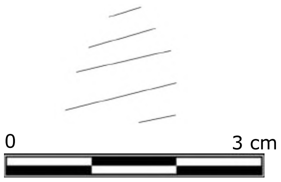 | NA | NA | between 65.5 ±4.8 ka and 59.4 ±4.6 ka | Nel & Haaland 2023, fig. 4 |
| K15 | 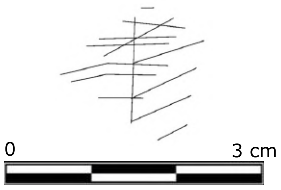 | NA | NA | between 65.5 ±4.8 ka and 59.4 ±4.6 ka | Nel & Haaland 2023, fig. 4 |

| | | | | | |
|---|---|---|---|---|---|
| K16 | 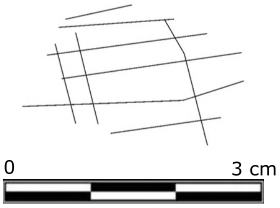  | NA | NA | between 65.5 ±4.8 ka and 59.4 ±4.6 ka | Nel & Haaland 2023, fig. 4 |
| K18 | 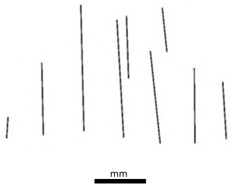 | NA | PPD | 64.6 ± 4.2 | Henshilwood et al. 2014, fig. 10f |

## Apollo 11

| ID | Tracing | publ. ID | Layer/SU | Date (ka BP, Jacobs et al. 2008) | Reference |
|---|---|---|---|---|---|
| A1 | 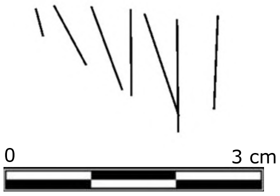 | NA | NA | 63 ± 2 | Ossendorf, Vogelsang 2023 fig. 4 |
| A2 | 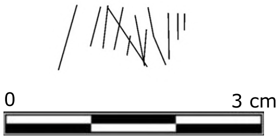 | NA | NA | 63 ± 2 | Ossendorf, Vogelsang 2023 fig. 4 |
