# Supplementary material for: Earliest geometries: A cognitive investigation of Howiesons Poort engraved ostrich eggshells
Source: PLoS One. 2026 Feb 11;21(2):e0338509. doi: 10.1371/journal.pone.0338509 (PMC12893581; doi:10.1371/journal.pone.0338509)
Supplement: S2 Document — (PDF) [file pone.0338509.s002.pdf]

S2. Methods and Results

Here we outline the methods and results applied to the extraction and quantitative analysis of the geometric data derived from the engraved ostrich eggshell (EOES) fragments listed in S1 Table (Dataset of EOES). In addition, we describe the method used to model the operational procedure ('geometric grammar') underlying the construction of the EOES patterns.

Data extraction with QGIS

Methods

Images of the line engravings on the EOES were taken from publications and the retracing of the incisions was done using QGIS 3, using normalized polylines (lines composed by segments) in absence of clear change or directions. Using Analysis and Geoprocessing tools the following data was extracted:

1. *Parallelism*. A first table (CSV) is extracted dividing the polylines in the segments that compose them, with the following information:
    - segment ID,
    - polyline ID of the segment,
    - segment inclination (azimuth degrees).
2. *Line type*. Another table (CSV) is extracted, containing, for each polyline, the number of the segments it is composed, as it follows:
    - polyline ID,
    - nr. of segments.
3. *Angles*. Another table (CSV) is exported with the minimal angle degree at each intersection point, calculated as follows:
    - finding the intersection points between polylines,
    - isolating the polyline segments at the intersection points,
    - extracting intersection coordinates and the smallest angle (absolute degree rounded without decimals) using an SQL query.

Results

The dataset was derived from 109 engraved ostrich eggshell (EOES) fragments. The extracted data include the following Table:

TYPE	DESCRIPTION	QUANTITY (TOT.)
Line IDs	Nominal data identifying polylines	1275
Segment IDs	Nominal data recording individual segments composing polylines	1635
Line Intersection	Nominal data regarding the polyline pairs intersecting with each other	1405
Min Angle at Intersection	Smallest of the four angles created at an intersection	1405, ranging from 1°-90°
Segment Azimuth	Orientation of each segment relative to geographic North	1635, ranging from 1°-180°
Coordinates at Intersection	Spatial coordinates of each intersection point	1405 coordinates (WGS84)

Analysis 1: Distribution of line type, parallelism, intersections, and right angles in the whole dataset

Methods

Frequencies and proportions of line types (straight vs. non-straight), parallel segments, line intersections, and angular degrees at intersections were computed using formulas and descriptive statistical tools built in Microsoft Excel. *T-tests,* R^2 and *p-values* were calculated using the Analysis *ToolPak*.

Results

Line type. A total of 1275 lines were drawn from 109 fragments (Figure 1). Of these, 1006 (78.90%) are straight and 269 (21.1%) are non-straight. The average number of non-straight lines per fragment is 3.95 (StdDev 5.13) with the highest counts observed on fragments D98 (n = 33), D49 (n = 21), and D58 (n = 19). For straight lines, the average per fragment is 9.31 (StdDev 7.93).

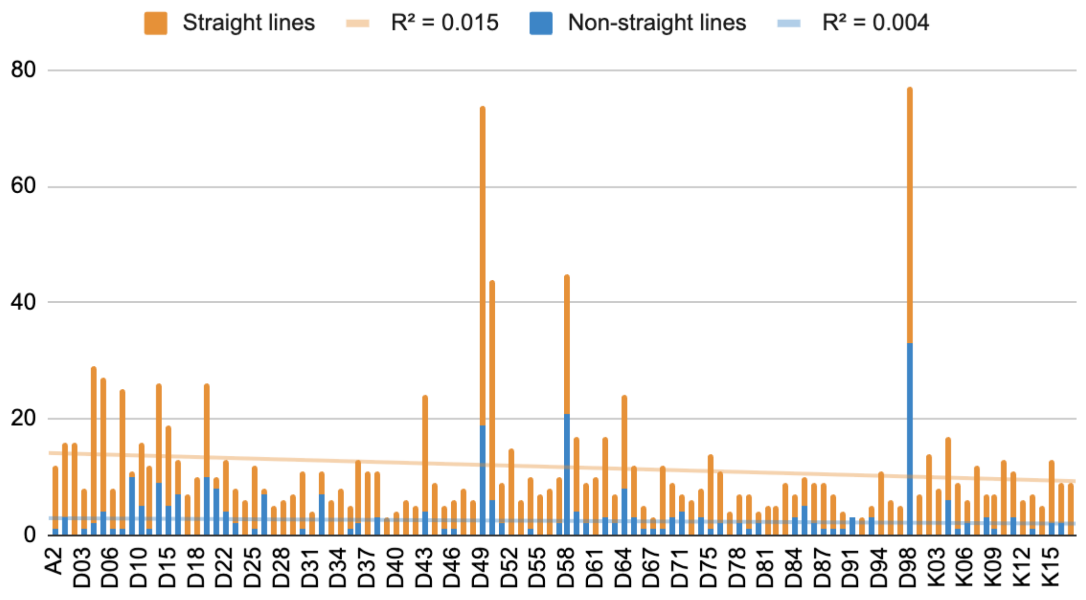

Figure 1. Distribution of straight and non-straight lines per fragment.

Parallelism. Out of 1635 segments, 1363 (83.4%) have at least one parallel, distributed across 107 fragments (Figure 2). Two fragments (D89 and D90) present no parallel segments. The average number of parallel segments per fragment is 12.73 (StdDev 17.22). The highest counts were recorded on fragments D98 (n = 126), D49 (n = 90), and D58 (n = 86), while seven fragments (D31, D39, D40, D67, D81, D82, D92) have only one pair of parallel segments each.

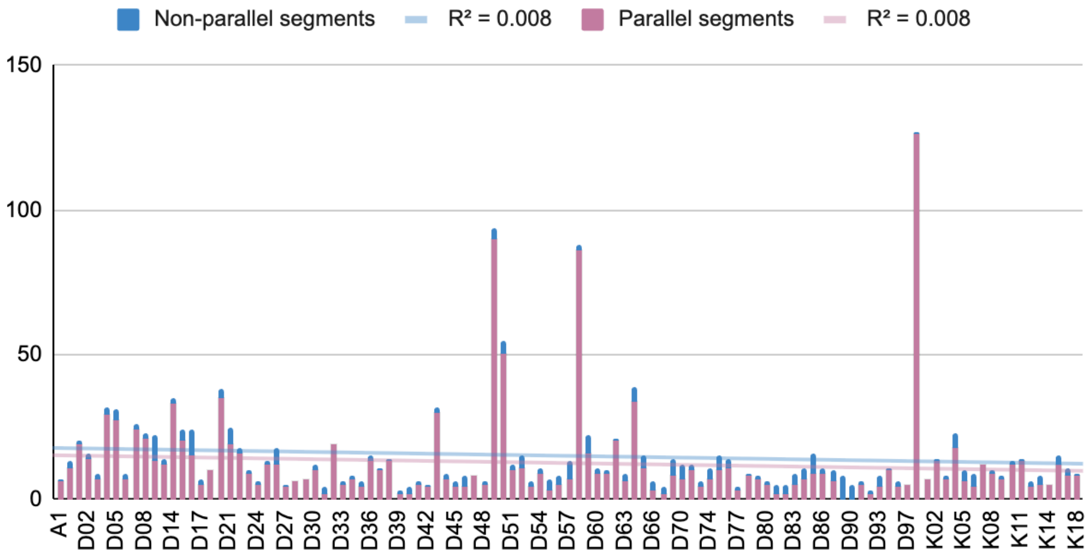

Figure 2. Distribution of parallel and non-parallel lines per fragment.

<u>Intersections.</u> A total of 1405 intersections were detected from 103 fragments (Figure 3). Six fragments (D17, D86, D91, K08, K14, K18) have no line intersections. The average number of intersections per fragment is 12.91 (StdDev 21.82). The most frequent intersections were found on D58 (n = 114), D98 (n = 104), D49 (n = 96), and D19 (n = 95). Conversely, six fragments (A01, D03, D22, D63, D67, D85) present a single intersection each.

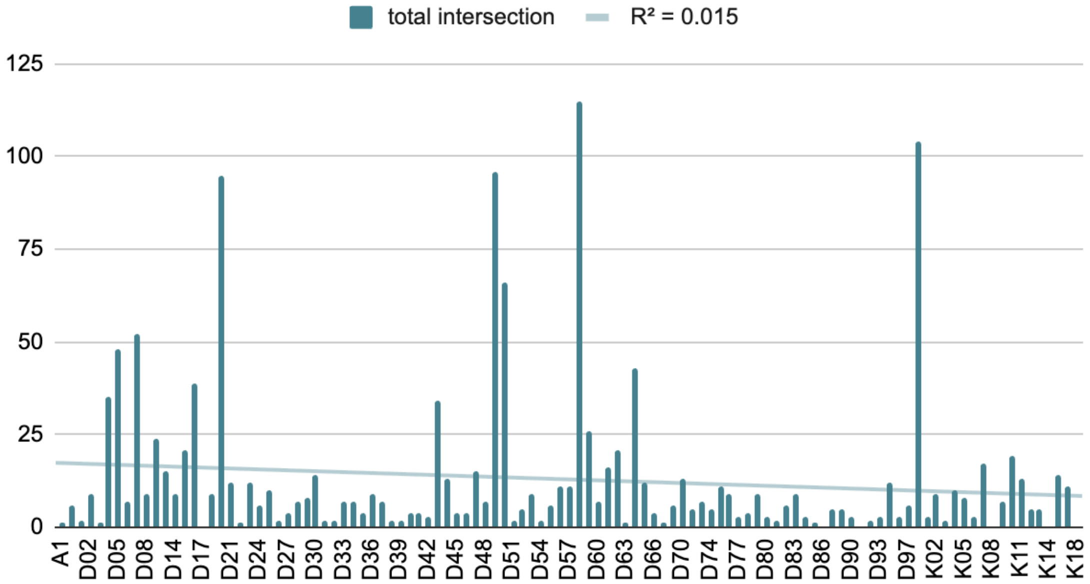

Figure 3. Distribution of number of intersections per fragment.

<u>Right angles.</u> The distribution of minor intersecting angles shows a tendency towards values close to 90° degrees (mean 66.87; StdDev 23.72, Skew -1.18; see Figure 4). The most frequent values are 87° (n = 74), followed by 84° (n = 71), and 86° (n = 65). The spread for all angles within each fragment (that is, angle difference between max & min values) varies greatly, ranging from 0° to 83°. This large spread is correlated with the number of intersections (r. 0.55; independent t(103)= 1.971, *p*= 0.013), suggesting that wider angular variability is influenced by greater intersection density.

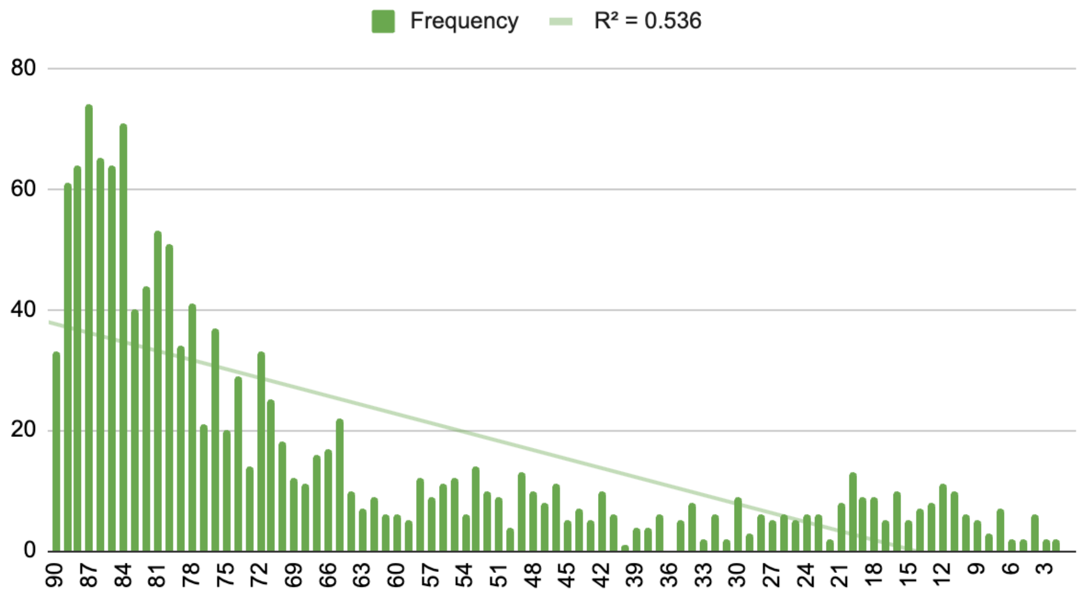

Figure 4. Distribution of min angle degrees across the entire dataset.

Analysis 2: Alignment strategies and angular relationships through multiple regression with residual analysis

Methods

Analysis 2 consists of two study phases: 1) grouping elements showing the same geometric feature (i.e. parallelism and angle degrees); 2) multiple regressions and residual analyses for highlighting internal regularities of the geometric values derived from previous grouping phase.

Two complementary grouping strategies were carried out for detecting regularities in alignment and angle degree: 1) parallelism grouping, which clusters segments with similar orientations (within ±3.5° azimuth difference, accounting for circularity around 0°/180°), capturing repeated directional alignment; and 2) minor angles grouping, which clusters the min angles at intersections (within ±3.5°), revealing consistent angular relationships such as near-right angles, repeated acute joins, mirrored symmetries, or patterned angular transitions. Each group was labeled sequentially (e.g., G1, G2...) and associated with the number of components it contains (e.g., fragment A1 = G1 [3], G2 [3] for parallelism, and fragment D02 = G1 [7], G2 [2] for angular relationships). For each group, we calculated its angular spread, defined as the difference between the smallest and largest azimuth values within the group for parallelism, and the difference between the smallest and largest angles for minor angles. Low spreads indicate highly consistent alignment and geometric regularity, whereas high spreads suggest looser internal organization in terms of parallelism and variability in intersection behavior. For parallelism, all 107 fragments had at least one group. For minor angles, 17 out of 103 fragments (16.5%) were excluded because had no grouping, while the remaining 86 (83.5%) presented at least one group.

The resulting group and spread data were then analyzed using multiple linear regression with residual analysis, calculated using the R Stats package (3.6.2). This statistical approach tests whether the maximum angular spreads – our proxy for internal regularity – can be predicted by two independent variables: the number of structural components (segments or intersections) and the number of internal groups. The generalized regression model is as follows:

$$max\_spread_i=\beta_0 + \beta_1 \cdot components_i + \beta_2 \cdot groups_i + \varepsilon_i$$

Where:

- max_spread_a = maximum angular spread for fragment *i*
- $components_a$ = number of segments (for parallelism) or intersections (for minor angles)
- $groups_a$ = number of parallel or angular groups
- ε_a = residual (error term)

Residuals were analyzed to assess model fit and identify outliers, that are fragments whose angular spread deviated significantly from predicted values, revealing compositional anomalies or exceptional regularity.

3.2. Results

Parallelism. The regression yields a strong model fit:

Multiple $R^2 = 0.736$, Adjusted $R^2 = 0.731$

$F(2, 106) = 147.8$, $p < 2.2e-16$, indicating high explanatory power

The intercept ($\beta_0 = 3.413$) represents the expected max spread when both predictors are zero. Although this is a hypothetical baseline (since all fragments have at least one segment), it anchors the model and supports interpretation of the coefficients. In terms of predictors:

- Total number of parallel segments is a strong and highly significant predictor ($\beta_1 = 0.384$, $p < 2e-16$), showing that higher segment count is correlated with increased angular spread.
- Number of groups, interestingly, has a negative coefficient ($\beta_2 = –0.734$, $p = 0.0102$), suggesting that a more structured subdivision of segments is associated with tighter angular alignment.

The residual standard error is 3.823, and most fragments fall within ±2.3° of predicted values. The residual analysis results indicate that, out of 107 fragments, 98 (91.59%) showed spread values that closely match model predictions (Figure 5). The remaining 9 fragments were identified as outliers: positive residuals (D14, D52, D58, D62, D65, K04) have higher spreads than predicted; negative residuals (D49, D50, D98), instead, display a degree of angular precision that exceeds statistical predictions.

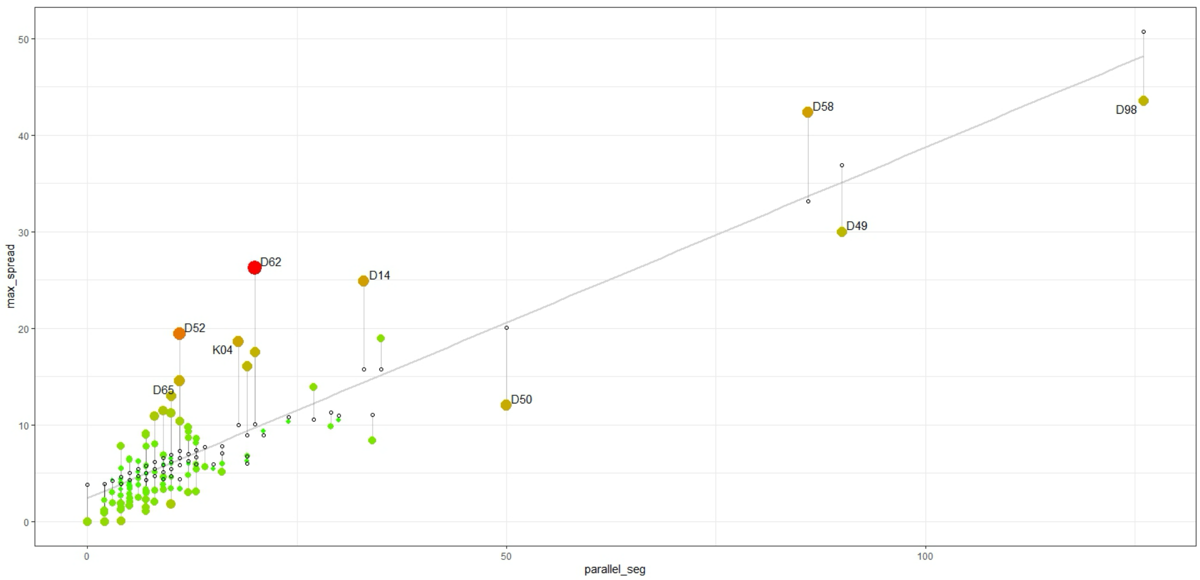

Figure 5. Scatterplot of the residual analysis for parallelism, outliers are labelled (made with ggplot package [Wickham 2016] for R).

<u>Min Angles.</u> The regression results indicate that the model significantly explains variation in angular spread:

Multiple $R^2 = 0.6665$, Adjusted $R^2 = 0.6584$

$F(2, 100) = 82.93$, $p < 2.2e\text{-}16$, indicating a strong overall model fit.

The intercept ($\beta_0 = 3.752$) represents the expected max spread when both predictors are zero, providing a baseline for interpreting the influence of the independent variables as for parallelism. Among the predictors:

- The number of intersections is a highly significant predictor of angular spread ($\beta_1 = 0.269$, $p < 2e\text{-}16$), confirming that higher density in intersections tends to produce greater angular variation.
- The number of groups does not reach statistical significance ($\beta_2 = -0.263$, $p = 0.577$), meaning that angular organization, in terms of grouping of adjacent angles, does not systematically relate to overall angular variability.

Residual diagnostics show a well-behaved distribution, with a residual standard error of 4.248 and most fragments falling approximately ±2.5 to 2.6 standard deviations, that is, within ±10.6 degrees of their predicted values.

Residual results show that out of 86 fragments, 80 (93.02%) fit the model well, while 6 fragments are identified as outliers (Figure 6): positive residuals (D04, D10, D43, D59, D62) have larger spreads than predicted, and one negative residual (D49) displays more angular precision than expected.

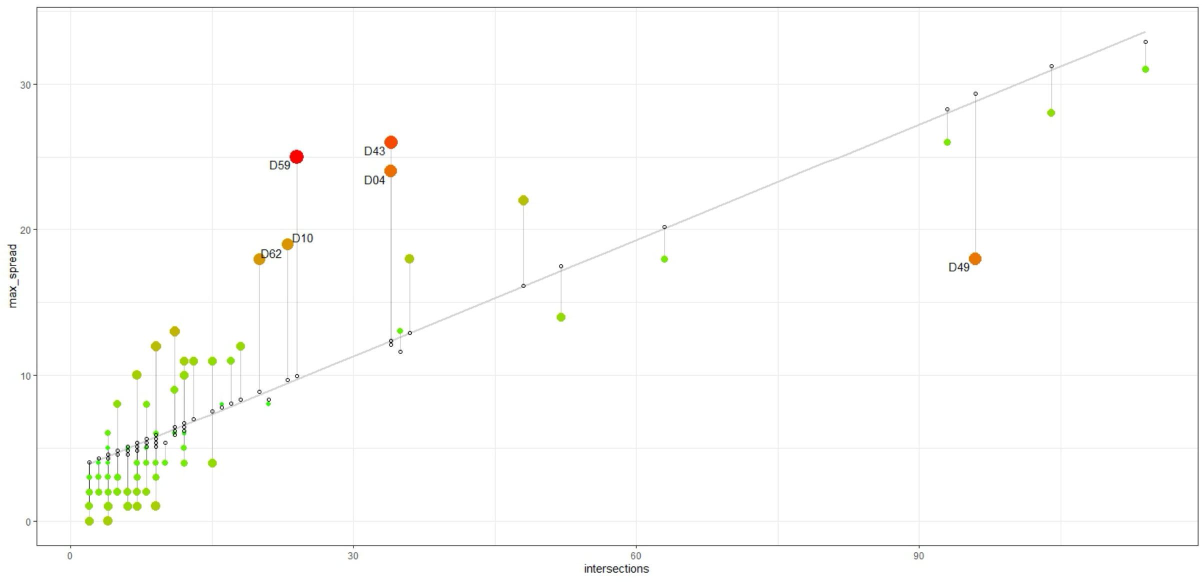

Figure 6. Scatterplot of the residual analysis for min angles, outliers are labelled (made with ggplot package [Wickham 2016] for R).

Analysis 3: Spatial analysis of intersections

Methods

Spatial analysis was performed by calculating Moran's I, using the Inverse Distance Weight (IDW) method. Given that the fragments already have a limited dimension (see S1 Table), no distance threshold was set for IDW.

Using QGIS 3, coordinates of intersection points were extracted, and their corresponding min angle degrees were calculated. To perform accurate spatial calculations, we converted the coordinates to a projected CRS (meters). Since the coordinates do not represent real-world location and our focus is on internal spatial relationships, we normalized and treated the distances as Euclidean. For this purpose, we used the Web Mercator projection (EPSG:3857) with the *Simple Feature* package for R.

We computed Moran's I for our spatial analysis using *Spatial Dependence* (spdep) library for R (Pebesma, Bivand, 2023; Bivand, 2022; Bivand & Wong, 2018; Bivand, Pebesma, Gómez-Rubio, 2013). Only fragments with a certain number of intersections ($n > 4$) can be considered in this study, to ascertain stable results for Moran's I. Then, to assess whether this index is significant ($p < 0.05$ indicates autocorrelation, e.g., clusters or scatters) or it is random, we performed a permutation test, to check the spatial correlation among the intersection points within each fragment. We dynamically adjusted the number of permutations according to the number of intersections in each fragment based on factorial ($n!$) and setting a minimum of 10 permutations, with a maximum of 999.

Results

The following graph (Figure 7) highlights the correlation between observed and expected Moran's I values. The grey line represents the 1:1 proportion between expectation (from permutations) and actual observation, with the points above the line showing positive spatial autocorrelation (e.g. clusters of similar angles detected inside the fragments). Fragments

laying on the line or below show no spatial autocorrelation, with distant internal values (e.g. alternating high and low angle degrees).

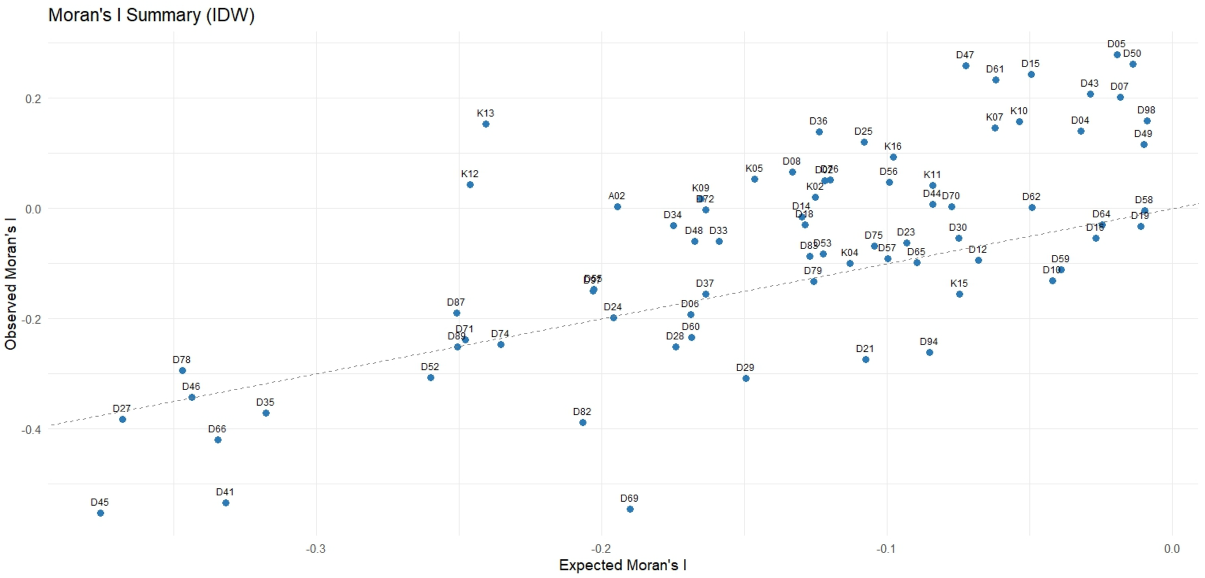

Figure 7. Scatterplot showing Moran’s I results for each individual fragment (made with ggplot package [Wickham 2016] for R).

20 fragments present $p < 0.05$ showing a consistency in spatial clusters of intersections having the same min angle. 47 fragments have a negative Moran’s I, which means they present spatial regularity but not correlated with the same angle degree. 20 fragments have a low *p-value* although two of them, D48 and D40, have respectively 7 and 4 intersections with negative or incalculable Moran’s I. The remaining fragments (n = 18) have all positive Moran’s I, which indicates a high level of autocorrelation in angles. 11 intersection angles present positive Moran’s I but a high *p-value*. These special cases are discussed in the paper. There is no correlation between Moran’s I and their *p-values* with the number of intersections. Indifferently, patterns with high or low number of intersections can have a significant patterning layout.

Analysis 4: Principal Component Analysis (PCA)

Methods

The dataset was built considering the following 4 variables for each fragment: nr. right angles/nr. intersections (‘right_angles’), nr. parallel segments/segments tot. (‘parallels’), nr. intersections/segments (‘intersections’), and Moran’s I (‘moran_I’). These values were normalized between 0 and 1. To handle missing values from Moran’s I and right angles (in fragments with no intersection points), VIM 6.2.2 (Templ et al., 2019; 2012; 2011), MissMDA 1.19 (Josse & Husson, 2016) and naniar 1.1 (Tierney & Cook, 2023) R packages were used to visualize, estimate the number of components from incomplete data and impute the missing values (see Figure 8). FactoMineR 2.9 (Lê et al., 2008) has been used to calculate and display PCA results.

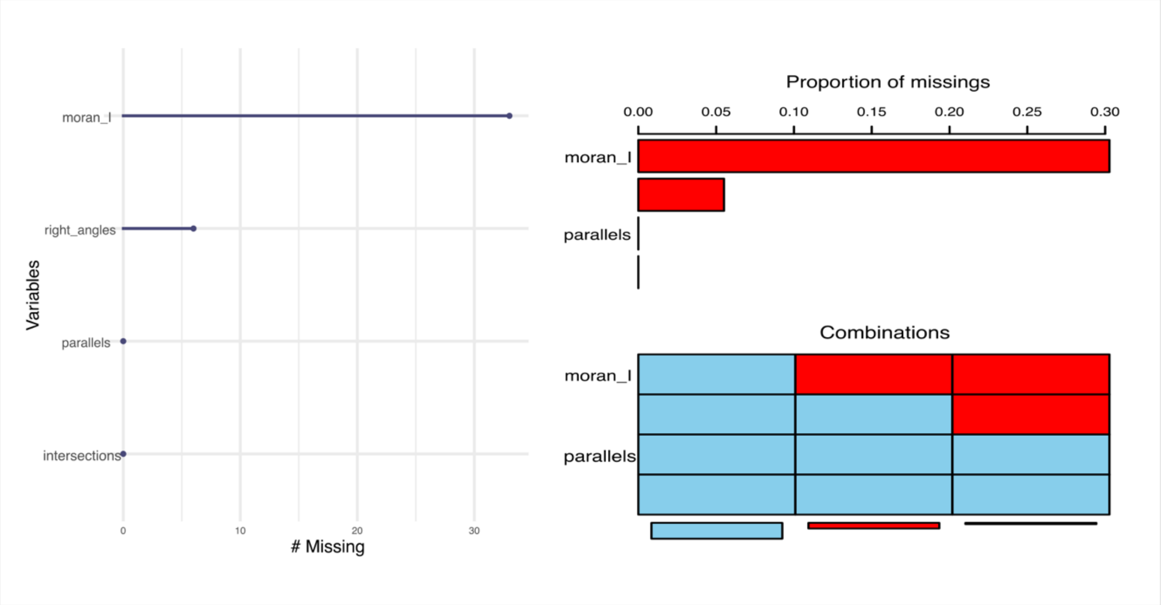

Figure 8. Proportion and aggregation of missing values to check how many of them are missing simultaneously (graph created with VIM package [Templ et al. 2019] for R).

Results

PCA was computed by using the dataset filled with the imputed data for the missing values. Its result shows the following eigenvalues:

	Dim.1	Dim.2	Dim.3	Dim.4
Variance	0.126	0.065	0.028	0.014
% of var.	54.149	27.850	12.021	5.980
Cumulative % of var.	54.149	81.999	94.020	100.000

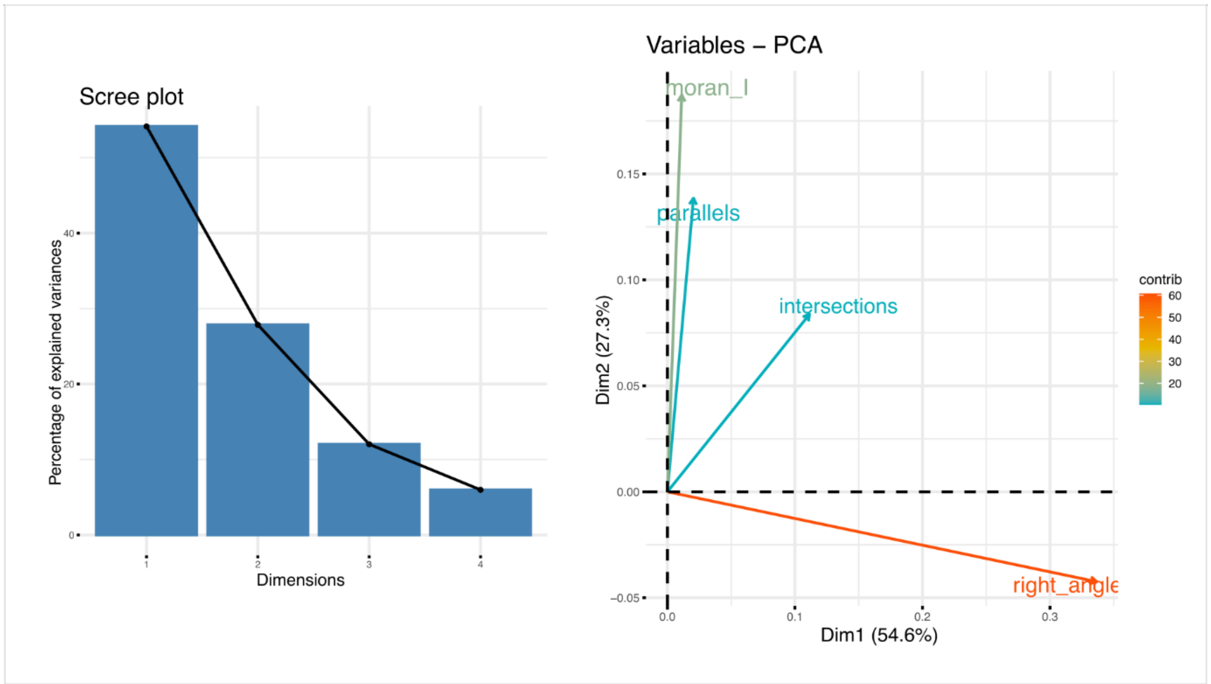

Figure 9. On the left, a scree plot of the explained variables (%) (generated with factoextra R package [Kassambara & Mundt, 2020]). On the right, a graph showing the variables contribution in PCA (graph designed with factoextra package [Kassambara & Mundt, 2020]).

Variables contributed to the principal components (Dim.1 and Dim.2) as follows:

	Dim.1	ctr	cos2	Dim.2	ctr	cos2	Dim.3	ctr	cos2
right_angles	0.334	88.702	0.962	-0.056	4.805	0.027	0.027	2.535	0.006
parallels	0.024	0.473	0.017	0.129	25.608	0.464	0.129	59.204	0.463
moran_I	0.027	0.573	0.016	0.192	56.772	0.837	-0.032	3.645	0.023
intersections	0.114	10.253	0.351	0.091	12.816	0.226	-0.098	34.616	0.263

Therefore (Figure 9), the number of right angles heavily contributes to the first principal component (Dim.1), with high contribution (ctr) and high representation (cos2). This means this value strongly influences the primary direction of variability. The nr. of parallels and Moran’s I are important in Dim.2 as both variables show high ctr and cos2 in the second principal component. This means that spatial autocorrelation (Moran’s I) and parallel structures are crucial in defining this direction of variability. While intersections contribute somewhat to all components, their influence is not concentrated in a single dimension but rather spread across dimensions. Dim.3 has a strong presence of parallels and intersections, meaning they introduce another independent way of structuring the data.

The biplot (Figure 10) arranges the fragments accordingly: a strong influence of right angles on PC1 (left-right) and a vertical spread driven by parallels and Moran’s I (PC2, top-bottom).

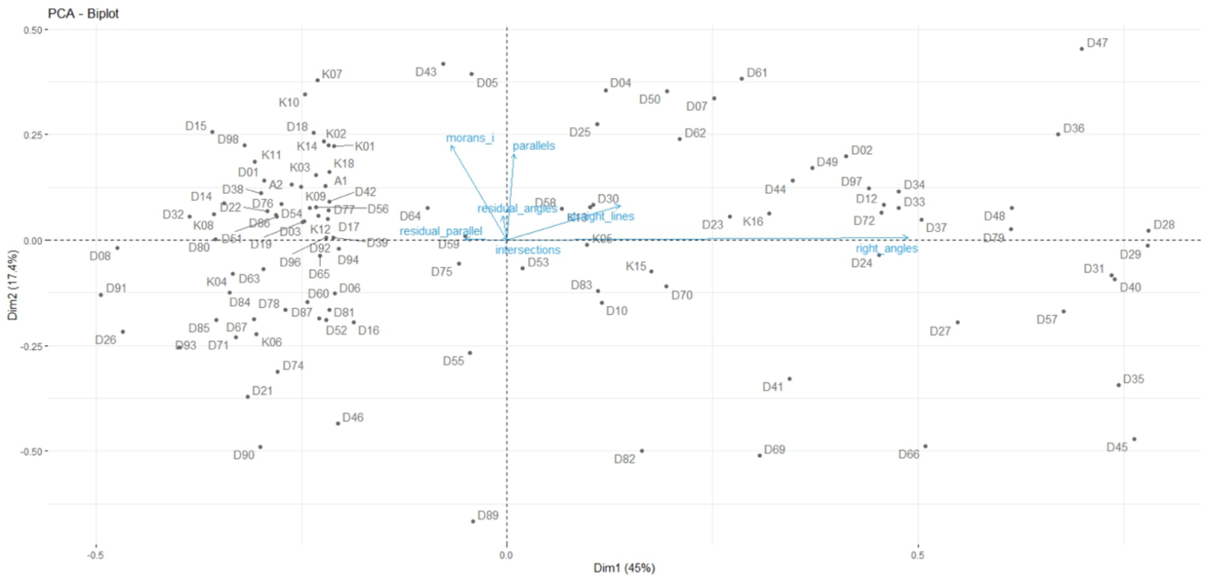

Figure 10. Biplot with variables showing the collocation of each individual fragment (made with FactoMineR 2.9 [Lê et al., 2008]) to match the category assigned in the dendrogram (Figure 11).

The agglomerative hierarchical cluster (Figure 11) displays groupings of fragments based on combinations of the variables, according to their values (none = 0 or N/A, low = 0.01-0.30, medium = 0.30-0.60, high = 0.60-1), as displayed in the table below. Cells are colored with a

	yellow (61.4%)	red (32.7%)	blue (15.26%)	grey (9.81%)
nr. right angles	none	low-to- high	none-to-medium	medium-to-high
Moran's I	none-to-high	medium-to-high	none-to-high	none-to-medium
parallelism	low-to-high	medium-to-high	none-to-high	medium-to-high
nr. intersections	none-to-medium	low-to-high	low-to-high	low-to-medium

green gradient if StdDev ≤ 0.15 (darker gradient for lower values).

The dendrogram (Figure 11) shows how these different categories (colors) combine to form clusters.

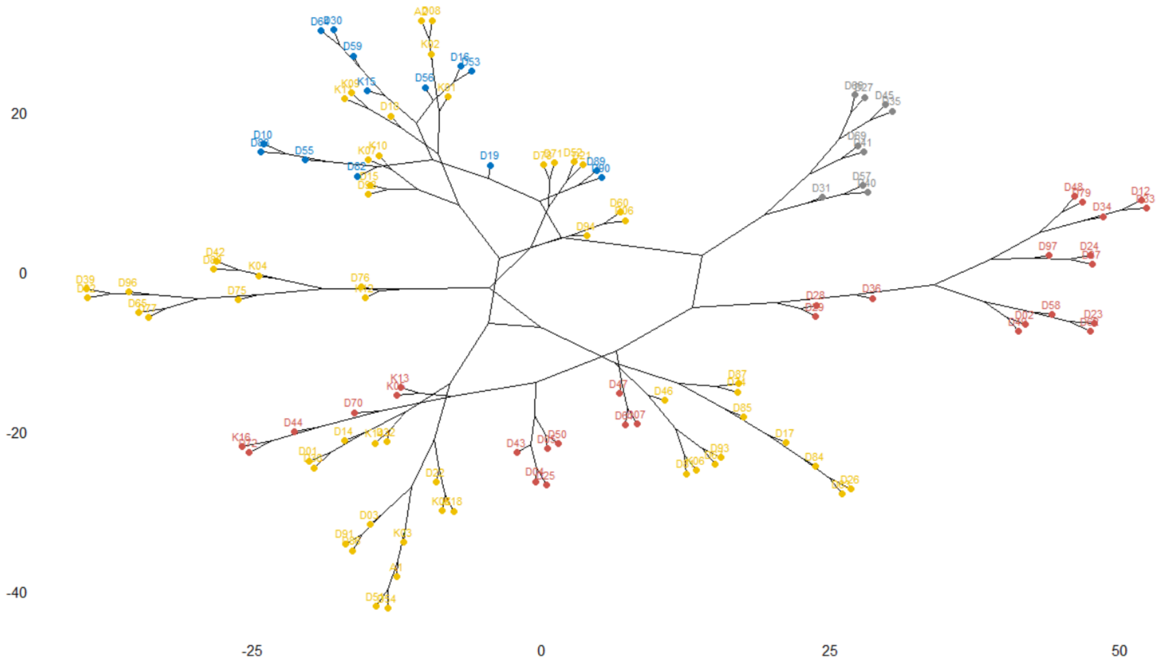

Figure 11. Tree showing the clusters per color and how they relate to each other (dendogram designed with factoextra and igraph 1.2.4 [Csárdi & Nepusx 2006; Csárdi et al. 2025]).

Method to extract the 'geometric grammar'

This pseudo-code (plain-language algorithm) outlines the fundamental operations underlying the construction of the engravings, as well as the core geometric features used. It also illustrates the hierarchical processes involved in generating the most complex EOES patterns.

```
#operations
ROTATE = move a feature around a point x with attribute: inclination = y°
TRANSLATE = move a feature to a distance x with attributes:
      inclination = same, size = same
ITERATE = repeat operations/features in the same hierarchical level
EMBED = nesting operations/features on additional hierarchical level

#basic geometric features
LINE = attributes: is_straight, length, inclination
ANGLE = create feature (
      given LINE a
      ITERATE + ROTATE a to create LINE b)
      with attribute: width;
PARALLEL = ITERATE LINE a to create LINE b (inclination = same as a)
ITERATE LINE b (inclination = a)
#create hatched band
Inside defined space (defined as:
   draw a LINE a,
   move to distance x != 0,
   draw a LINE b PARALLEL to a)
EMBED a LINE c (length = from LINE a to LINE b, inclination = y)
      and while there is still enough space in defined space, ITERATE:
            TRANSLATE the previous LINE to a distance z

#create diamond shape
Inside defined space (defined as polygon with attributes: height, length)
EMBED a LINE a (length = height of defined space, inclination = x°)
      and while there is still enough space in length of defined space,
      ITERATE:
            TRANSLATE the previous LINE to a distance z
and EMBED a LINE b (length = height of defined space,
            inclination = +- 50° with respect to LINE a)
      and while there is still enough space in length of defined space,
      ITERATE:
            TRANSLATE the previous LINE to a distance z

#create grid
Inside a defined space (defined as:
   draw a LINE a
   ITERATE N times:
      TRANSLATE the previous LINE to a distance x)
EMBED a LINE b (inclination = +- 90° with respect to LINE a)
      and while there is still enough space in length of defined space,
      ITERATE:
            TRANSLATE the previous LINE to a distance z
```

References

Bivand R. (2022). “R Packages for Analyzing Spatial Data: A Comparative Case Study with Areal Data.” *Geographical Analysis*, 54(3), 488-518. doi:10.1111/gean.12319.

Bivand R, Pebesma E, Gómez-Rubio V (2013). *Applied spatial data analysis with R, Second edition*. Springer, NY.

Bivand R, Wong D (2018). “Comparing implementations of global and local indicators of spatial association.” *TEST*, 27(3), 716–748. doi:10.1007/s11749-018-0599-x.

Csárdi G, Nepusz T (2006). “The igraph software package for complex network research.” *InterJournal*, Complex Systems, 1695.

Csárdi G, Nepusz T, Traag V, Horvát Sz, Zanini F, Noom D, Müller K, Schoch D, Salmon M (2025). *igraph: Network Analysis and Visualization in R*. doi:10.5281/zenodo.7682609

Josse, J., & Husson, F. (2016). missMDA: a package for handling missing values in multivariate data analysis. *Journal of statistical software*, 70, 1-31.

Kassambara, A. and Mundt, F. (2020) Factoextra: Extract and Visualize the Results of Multivariate Data Analyses. R Package Version 1.0.7. https://CRAN.R-project.org/package=factoextra

Lê S, Josse J, Husson F (2008). “FactoMineR: A Package for Multivariate Analysis.” *Journal of Statistical Software*, 25(1), 1–18. doi:10.18637/jss.v025.i01.

Pebesma E, Bivand R (2023). *Spatial Data Science With Applications in R*. Chapman & Hall.

Templ, M., Kowarik, A., Alfons, A., de Cilia, G., Prantner, B., & Rannetbauer, W. (2019). *Visualization and imputation of missing values*. Springer.

Tierney N, Cook D (2023). “Expanding Tidy Data Principles to Facilitate Missing Data Exploration, Visualization and Assessment of Imputations.” *Journal of Statistical Software*, 105(7), 1–31. doi:10.18637/jss.v105.i07.

Templ M., Alfons, A., Filzmoser, P. (2012) Exploring incomplete data using visualization tools. *Journal of Advances in Data Analysis and Classification*, Online first. DOI: 10.1007/s11634-011-0102-y.

Templ, M., Kowarik, A., Filzmoser, P. (2011) Iterative stepwise regression imputation using standardand robust methods. *Journal of Computational Statistics and Data Analysis*, Vol. 55, pp. 2793-2806.

Wickham, H (2016). ggplot2: Elegant Graphics for Data Analysis. Springer-Verlag New York.
