# Supplementary material for: Earliest geometries: A cognitive investigation of Howiesons Poort engraved ostrich eggshells
Source: PLoS One. 2026 Feb 11;21(2):e0338509. doi: 10.1371/journal.pone.0338509 (PMC12893581; doi:10.1371/journal.pone.0338509)
Supplement: Attachment — Submitted filename: Response to Reviewers.docx [file pone.0338509.s004.docx]

**PONE-D-25-36789**

**Earliest geometries. A cognitive investigation of Howiesons Poort engraved ostrich eggshells**

**PLOS ONE**

Dear Iris Groman-Yaroslavski,

Dear Editors,

Thank you for your assistance and for sharing the information regarding the journal requirements and reviewer comments. Please find our responses below (in **blue**), addressing both the journal requirements and the reviewers’ comments.

**Journal requirements**

When submitting your revision, we need you to address these additional requirements.

1. Please ensure that your manuscript meets PLOS ONE's style requirements, including those for file naming. The PLOS ONE style templates can be found at <https://journals.plos.org/plosone/s/file?id=wjVg/PLOSOne_formatting_sample_main_body.pdf> and [https://journals.plos.org/plosone/s/file?id=ba62/PLOSOne_formatting_sample_title_authors_affiliations.pdf](https://track.editorialmanager.com/CL0/https:%2F%2Fjournals.plos.org%2Fplosone%2Fs%2Ffile%3Fid=ba62%2FPLOSOne_formatting_sample_title_authors_affiliations.pdf/1/010f019970918b92-4e47f551-373d-4040-a077-873073e90f20-000000/ZlY8iENz5-A5lMtPa3m0AI7Fo8vzy3zJYNFpuyUkwZA=229)

**We confirm that our revised manuscript fully meets PLOS ONE’s style and file-naming requirements.**

2. In your manuscript, please provide additional information regarding the specimens used in your study. Ensure that you have reported human remain specimen numbers and complete repository information, including museum name and geographic location. If permits were required, please ensure that you have provided details for all permits that were obtained, including the full name of the issuing authority, and add the following statement: *'All necessary permits were obtained for the described study, which complied with all relevant regulations.*' If no permits were required, please include the following statement: *'No permits were required for the described study, which complied with all relevant regulations.*' For more information on PLOS One's requirements for paleontology and archeology research, see [https://journals.plos.org/plosone/s/submission-guidelines#loc-paleontology-and-archaeology-research](https://track.editorialmanager.com/CL0/https:%2F%2Fjournals.plos.org%2Fplosone%2Fs%2Fsubmission-guidelines%23loc-paleontology-and-archaeology-research/1/010f019970918b92-4e47f551-373d-4040-a077-873073e90f20-000000/15QiPp7WEpquQQUEhFi3x6kUSEJv_v3Ywq89AxqRQQw=229).

**We confirm that our study is based exclusively on previously published materials. No new excavation, handling, or analysis of human remains or archaeological specimens was conducted.**

3. We note that the grant information you provided in the ‘Funding Information’ and ‘Financial Disclosure’ sections do not match. When you resubmit, please ensure that you provide the correct grant numbers for the awards you received for your study in the ‘Funding Information’ section.

**Thank you. We have verified the information and corrected the grant details to ensure consistency between the Funding Information and Financial Disclosure sections. The correct grant numbers are now provided in the Funding Information section.**

4. We note that Figure 1 in your submission contain [map/satellite] images which may be copyrighted. All PLOS content is published under the Creative Commons Attribution License (CC BY 4.0), which means that the manuscript, images, and Supporting Information files will be freely available online, and any third party is permitted to access, download, copy, distribute, and use these materials in any way, even commercially, with proper attribution. For these reasons, we cannot publish previously copyrighted maps or satellite images created using proprietary data, such as Google software (Google Maps, Street View, and Earth). For more information, see our copyright guidelines: <http://journals.plos.org/plosone/s/licenses-and-copyright>.

We require you to either (1) present written permission from the copyright holder to publish these figures specifically under the CC BY 4.0 license, or (2) remove the figures from your submission:

4.1. You may seek permission from the original copyright holder of Figure 1 to publish the content specifically under the CC BY 4.0 license. We recommend that you contact the original copyright holder with the Content Permission Form (<http://journals.plos.org/plosone/s/file?id=7c09/content-permission-form.pdf>) and the following text: *“I request permission for the open-access journal PLOS ONE to publish XXX under the Creative Commons Attribution License (CCAL) CC BY 4.0 (*[*http://creativecommons.org/licenses/by/4.0/*](http://creativecommons.org/licenses/by/4.0/)*). Please be aware that this license allows unrestricted use and distribution, even commercially, by third parties. Please reply and provide explicit written permission to publish XXX under a CC BY license and complete the attached form.”* Please upload the completed Content Permission Form or other proof of granted permissions as an "Other" file with your submission. In the figure caption of the copyrighted figure, please include the following text: “Reprinted from [ref] under a CC BY license, with permission from [name of publisher], original copyright [original copyright year].”
4.2. If you are unable to obtain permission from the original copyright holder to publish these figures under the CC BY 4.0 license or if the copyright holder’s requirements are incompatible with the CC BY 4.0 license, please either i) remove the figure or ii) supply a replacement figure that complies with the CC BY 4.0 license. Please check copyright information on all replacement figures and update the figure caption with source information. If applicable, please specify in the figure caption text when a figure is similar but not identical to the original image and is therefore for illustrative purposes only.

The following resources for replacing copyrighted map figures may be helpful:

USGS National Map Viewer (public domain): <http://viewer.nationalmap.gov/viewer/>

The Gateway to Astronaut Photography of Earth (public domain): <http://eol.jsc.nasa.gov/sseop/clickmap/>

Maps at the CIA (public domain): <https://www.cia.gov/library/publications/the-world-factbook/index.html> and <https://www.cia.gov/library/publications/cia-maps-publications/index.html>

NASA Earth Observatory (public domain): <http://earthobservatory.nasa.gov/>

Landsat: <http://landsat.visibleearth.nasa.gov/>

USGS EROS (Earth Resources Observatory and Science (EROS) Center) (public domain): [http://eros.usgs.gov/#](http://eros.usgs.gov/)

Natural Earth (public domain): [http://www.naturalearthdata.com/](http://track.editorialmanager.com/CL0/http:%2F%2Fwww.naturalearthdata.com%2F/1/010f019970918b92-4e47f551-373d-4040-a077-873073e90f20-000000/heGcovKVBzn6W35wpQWYq6HEBuR62zPi6Y22R47xUAI=229)

**We have a new Figure 1 that meets PLOS ONE’s requirements. This version was created in QGIS using as basemap CARTO, © OpenStreetMap contributors, CC BY 4.0 and it will be referenced in the caption.**

**Also, we have revised the supplementary material 'S1 Table', removing all copyrighted content to ensure full compliance with publication and licensing requirements.**

5. If the reviewer comments include a recommendation to cite specific previously published works, please review and evaluate these publications to determine whether they are relevant and should be cited. There is no requirement to cite these works unless the editor has indicated otherwise.

**N/A.**

While revising your submission, please upload your figure files to the Preflight Analysis and Conversion Engine (PACE) digital diagnostic tool, [https://pacev2.apexcovantage.com/](https://track.editorialmanager.com/CL0/https:%2F%2Fpacev2.apexcovantage.com%2F/1/010f019970918b92-4e47f551-373d-4040-a077-873073e90f20-000000/2juFHfMQRVcT1_k_7MDSnZwLIqYnhv3xyZ_qMffFk8k=229). PACE helps ensure that figures meet PLOS requirements. To use PACE, you must first register as a user. Registration is free. Then, login and navigate to the UPLOAD tab, where you will find detailed instructions on how to use the tool. If you encounter any issues or have any questions when using PACE, please email PLOS at figures@plos.org. Please note that Supporting Information files do not need this step.

**We have uploaded all figure files (Figures 1–9) to the PACE tool, as requested, and updated them to meet PLOS requirements. Please note that:**

- **Figure 1 has been completely redone;**
- **Figure 7 is a newly added figure;**
- **Figure 8 (previously Figure 7) has been edited, with minor textual modifications;**
- **Figure 9 (previously Figure 8) has been updated accordingly.**

**Response to Reviewers**

We thank the reviewers for their insightful comments which have given us the opportunity to significantly improve our paper. We agree with the reviewers on the necessity of revising the manuscript to better reflect the importance of our study and to highlight its cognitive perspective. We significantly reviewed the manuscript accordingly, in order to tie the sections harmoniously, through major adjustments and insertions. Our responses (in **blue** below) address and explain our changes, and report the specific lines in the manuscript which we have modified. In the manuscript, we have kept the revisions in track changes.

**Reviewer #1**

General remarks – the manuscript presents in intriguing idea, and interpretive framework for the early geometric patterns of EOES. Yet the large theoretical background described in the abstract and introduction do not seem to be validated, at least not as described in the conclusions. It is possibly not well explained, but the conclusions seem to be more “down to earth” compared to what is presented in the introduction. Thus, there seems to be a disconnect between the introduction and the discussion, which should be readjusted to focus more on the cognitive operations, and the transmission and transformation of the geometric forms.
 In general, there is tendency for over-complication in terms of language, I would recommend careful read through of the manuscript to simplify words and restructure complicated sentences.
 Overall, this could be a very interesting paper, after major revisions, simplifying the language and ideas, clarifying the methodology, and tying the theoretical background to the practical work.

**We are grateful for this constructive comment. We have substantially rewritten the manuscript to improve clarity, simplify language, and strengthen alignment between theoretical framing and the results. The introduction, in particular, has been refocused on the cognitive operations that can be empirically reconstructed, and the discussion and conclusion now explicitly address these operations and the results that support them. We have striven to tie the sections of the manuscript together, and clarified the overall structure, with heavy interventions in the conclusions as well. Moreover, we revised the titles of the three subsections in the ‘*Results and discussion*’ section to better connect the statistical analyses to the cognitive implications. The new titles are as follows: ‘*Distribution of geometric features: the salience of parallelism and orthogonality*’ (originally ‘*Distribution of geometric features and their salience in the whole dataset*’), ‘*Regularities in alignment strategies and angular patterns: toward a ‘geometric grammar’’* (originally ‘*Regularities in alignment strategies and angular patterns*’), and *‘Spatial arrangements of intersection geometries: regular distances’* (originally *‘Variations from regular patterns and their salience’*). We are confident that the manuscript in this latest version reflects the results we have reached and conveys clearly our conclusions.**

**Abstract**
 • The abstract is too general and theoretical and doesn’t really describe the essentials of the research. It should include the research question, the analytical tools used to tackle the question, as well a succinct description of the results and conclusions.

**Thank you, we have rewritten the abstract to describe more clearly the aims, methods, and results of our analysis.**

**Method**
 • There are several problems in the methodology, or points that could use clarification. It is understood that the data derives from published photographs, but this means that the authors have no control over positioning of the fragments. A non-uniform way of positioning will alter the lines, spatial relations between lines (parallelness etc.), the measurement of the angles, curvature, as well as distances between points. The authors acknowledge only the possible distortion in discussing metric measurements, but do not explicitly explain why they are measured anyway. If the measurements are skewed, can the information collected from them be crucial? The bias should be acknowledged for all parameters.

**We now explicitly explain in the manuscript that, although possible distorsions are inevitable when dealing with photographs, we have normalized lines working with non-accidental properties (NAPs). This is crucial as NAPs do not alter with changing viewpoints. Therefore this should strengthen the results via the method we used (Please see lines 359-364 in the manuscript):**

*‘We mitigated distortion issues, which are inevitable when working with second-hand material such as photographs, by visually normalizing the lines following their ‘non-accidental properties’ (NAPs) which are stable when viewpoint changes [32-35]. These can include curvature (a line is straight or curved), collinearity (edges align to form a continuous line), symmetry (consecutive or mirror balance), parallelism (edges remain equidistant), and co-termination (edges meet at a single point).’*

**Conversely, when measurements are included, which are instead susceptible to distortions, we applied empirically defined tolerance thresholds on angular deviations (for parallelism and orthogonality), and relational distance of points rather than absolute metrics for spatial organization. This is stated in the manuscript in lines 418-422:**

*‘Metric properties, as opposed to NAPs, vary continuously according to different viewpoints and are more susceptible to perceptual error (e.g., distance misestimation, angular openings [33,34]). Therefore, when classifying features, such as parallelism derived from line inclinations or right/non-right distinction derived from angular openings, we applied tolerance thresholds informed by empirical studies [35].’*

**This ensures that deviations are not only acknowledged, but taken into account and mitigated, and that non-accidental properties will in any case preserve perspective.**

• What does tracing “both manually and digitally” mean? (Line 122) It seems like manual tracing in a digital format (QGIS)

**We have clarified this point as suggested. Please see lines 358-359:**

*‘We manually traced the EOES motifs line by line using QGIS 3 (Fig 3), to extract their geometric and spatial features’.*

• “Visual normalization” is unclear, does this mean that the normalization is particular to each etched line, as opposed to normalizing the assemblage? Line 125 - Citation of Biederman 1987 – a short sentence explaining the idea and the relevance of the theory to the present research. It seems a crucial reference to understand the method but a reference alone is not enough.

**We very much appreciate the suggestion. We have refined the explanation on ‘visual normalization’ since what we have specifically focused on are Non Accidental Properties of the markings and explained them as indicated in the comment above.**

• In the methods section there is a list of numbered points (from line 129), which are not entirely clear. Do they refer to the tolerance thresholds? Or are they steps in the methodology? If so, it should be differentiated in the text as a sub-title within the methodology. The points could include references to figures or possibly replace by a figure illustrating and outlining the analytical steps; alongside an illustration of the differentiation between lines and segments (and their “directionality”) and examples of why and how they are segmented.

**We have implemented a series of changes throughout this subsection to clarify the definitions and methodological principles of our retracing of the EOES (the numbered points). Please see specifically lines 423-436:**

*‘We define and classify geometric features and measurements as follows:*

1. *Line type: straight vs. non-straight. A line is defined as straight if it connects two endpoints without deviation. A non-straight line includes at least one directional change, marked by a vertex joining two segments.*
2. *Parallelism: this is assessed at the segment level. Two segments are considered parallel if their inclinations differ by no more than ±3.5°. This threshold is supported by evidence that people can discriminate between angle differences as small as 7° with approximately 53% accuracy [35].*
3. *Intersection geometry: this is detected between crossing lines, and the minor angle (the smallest angle at the intersection) is extracted for angular categorization.*
4. *Angular opening: minor angles are classified as right vs. non-right, with a tolerance of 7° off 90° for defining right angles.*
5. *Spatial organization: distances between intersection points are calculated from coordinates to highlight possible spatial regularities within each fragment.’*

**Reviewer #2**:

This paper addresses an interesting question in the field of cognitive archaeology, that of what cognitive mechanisms underlie graphic symbolism in our species. The authors attempt to contribute to answering that question by analysing a corpus of early visual sign manifestations: the engraved ostrich eggshells (OES) found at the South African sites of Diepkloof and Klipdrft Shelter, and Apollo 11 in Namibia, all dating back some 60-65,000 years to an archaeological complex known as Howiesons Poort.
The present study carries out the first quantitative analyses of the OES with the aim to identify the cognitive processes that supported the creation of the engravings. Whereas the methods seem meticulous and raise important questions about the materials and their creators, I also found several shortcomings, detailed below.

**We thank the reviewer for this comment that highlighted an important point in our paper, and gave us the opportunity to elaborate and expand on this topic in the introduction. Please see lines 57-61:**

*‘The EOES have typically been discussed within the broader debate on behavioral modernity [6,7] and frequently included within the same symbolic ‘package’ as other early manifestations of abstract marking and symbolic material culture [8-11]. However, rather than focusing on their symbolic potential and interpretation, here we delve into a deeper analytical level, exploring the cognitive foundations that underlie the geometric construction of the engravings.’*

**We recognize the relevance of the topic in the current debate. However, our analysis is strictly tied to cognitive behaviour and operational strategies. We have advisedly avoided any investigation or debate into symbolism, because it would have been a fully loaded articulation of a discussion that is not germane to our specific focus. We however have added crucial references dedicated to this important issue in the manuscript.**

**1. General.**
One of the main claims of the study seems to be that the engraved OES show intentionality, awareness, and precise execution. This is backed by the precise quantitative analyses presented by the authors. However, the intentionality of the engravings has never been in question. Since they were first published, several papers have argued that the motifs were made purposefully and originally constituted complex geometric patterns (e.g., Henshilwood et al., 2014; Nel and Haaland, 2023; Texier et al., 2010; 2013; Tylén et al., 2020 Vogelsang et al., 2010). Also, the earlies Blombos engraving has already been identified as following geometric primitives. In this sense, I wonder what this paper contributes to the already ongoing discussion about early symbolism, other than another method of analysing the geometric patterns.

**Thank you for this comment. Our analysis does not aim to demonstrate or confirm intentionality and awareness, as this has already been established in other publications (Texier et al. 2010; Tylén et al., 2020). What we want to outline is that we can observe, through rigorous statistical methods, that the geometric features of the markings show a clear and complex cognitive architecture. We have accordingly clarified our aim at the beginning of the introduction as follows (please see lines 23-56 in the manuscript):**

*‘In this paper, we aim to investigate the cognitive processes underlying the construction of these patterns through the first method-based, quantitative assessment of their formal and measurable features. Indeed, these engravings have been classified into motif groups according to their ‘geometric (*sensu lato*)’ characteristics, based on direct visual inspection of the fragments [2]. But, to date, no dedicated study has statistically examined their formal geometry, a crucial aspect for understanding whether these patterns reflect genuine cognitive structuring.’*

**Not only do we observe complexity, but we also see that this complexity amounts to a regular and specific cognitive foundation that can be studied further, by analyzing the geometric characteristics of the EOES, measuring their features, and testing them statistically. This is a novel path of analysis. In previous publications, for instance Texier et al. 2013, the authors write about ‘geometric (*sensu lato*) patterns’ (Texier et al. 2013:3419). We intended here to study closely, via quantitative methods, this *sensu latu* for the first time. In fact, the only cognitive article published on the subject (Tylén et al., 2020) has a different aim tied to intentionality, memorability, and transmission, and uses a larger dataset. We have revised the introduction to clarify why (and how) our research fits into the scholarly debate and what aims we set out to reach.**

**2. Introduction**
The introduction goes into an interesting discussion on the cognitive fundaments of geometry and spatial cognition, but I feel this is not followed up in the discussion with sufficient depth. After having shown that the OES patterns were conceived following geometric ‘rules’, it would have been interesting to read why this is relevant to human cognition during the MSA.

**We understand the reviewer’s standpoint. To this end, we have fine-tuned the introduction in terms of the cognitive processes that lead *Homo sapiens* to combine and embed geometric ‘primitives’ and their relevance in the HP context. In the manuscript, we have clarified the theoretical framework to emphasize how Euclidean intuitions and their ruled-based combinations form part of human core knowledge, establishing a direct link between cognitive studies on geometric reasoning and the measurable parameters applied in our analysis of EOES engravings. Please see lines 62-77:**

*‘Our analysis builds on studies suggesting that flexible Euclidean intuitions are part of human core knowledge as a cross-cultural universal, together with spatial orientation and object recognition [12-18]. These intuitions include Euclidean geometric features, such as line, point, parallelism, and right angle, and are defined as ‘conceptual primitives of geometry’ [12]. Importantly, these primitives can be combined through a set of rule-based operations which can be iterative (insertion of additional elements within the same hierarchical level) or embedded (nested elements on additional hierarchical levels) [17-19].*

*Even if some Euclidean principles are ingrained in our cognitive system, they are occasionally violated in human perception [14]. Investigations on cognitive maps have found that our spatial thought rather rests on a hybrid Euclidean and non-Euclidean spatial representation [20]. Nonetheless, this hybrid space can be successfully projected on a Euclidean (2D) surface - and vice versa - thanks to a slight spontaneous distortion that aims to keep visual salience (the perceptual prominence or recognizability) of basic geometric features such as right angles and parallelism [21-24]. This perspective is crucial for our analysis, as it facilitates an investigation of the EOES through measurable geometric parameters.’*

**Also please see lines 81-85, which we modified as follows:**

*‘The results show a high degree of regularity in the arrangement of salient geometric features, involving procedural and hierarchical operations. What will become apparent is, thus, the emergence of a ‘geometric grammar’, that is a system of geometric rules through which* Homo sapiens *in the HP organized visual forms, revealing the cognitive foundations of structured graphic behavior.’*

**We are confident that the introduction is now clearer in aim and focus. Moreover, the relevance of the ‘geometric grammar’ is now explicitly addressed in the Conclusion. Please see lines 1081-1085:**

*‘Consequently, the EOES embody the cognitive potential and foundational principles underlying symbol making—the ability to iterate features, embed structures hierarchically, and maintain visual coherence through abstraction. These engravings therefore constitute an early material expression of complex graphic representation, attesting to a species-specific human capacity for organizing geometric thought.’*

**3. Materials and Methods**
 a) Regarding the materials and methods, it is understandable that the authors worked from published materials, and this practice is common in archaeology. However, I miss a limitations section where it is explicitly acknowledged that working from second hand sources has constraints, along with issues of working with fragmentary material, and the chronological span between samples, which I feel is much underplayed throughout the paper (despite all materials belonging to the HP, there are potentially five millennia between some of them).

**We agree that clarifications were needed and we have added them respectively in the ‘Retracing and segmenting’ subsection. Please see lines 359-364:**

*‘We mitigated distortion issues, which are inevitable when working with second-hand material such as photographs, by visually normalizing the lines following their ‘non-accidental properties’ (NAPs) which are stable when viewpoint changes [32-35]. These can include curvature (a line is straight or curved), collinearity (edges align to form a continuous line), symmetry (consecutive or mirror balance), parallelism (edges remain equidistant), and co-termination (edges meet at a single point).’*

**And in the ‘Contextualizing the EOES’ subsection (see lines 337-341):**

*‘However, we take the assemblage in its entirety, since the aim of this analysis is to study the cognitive underpinnings of the EOES: this approach allows us to capture overarching structural regularities. Methodologically, this choice is supported by the material, cultural, geographic, and chronological uniformity of the HP, which ensures that our comprehensive analysis is not hindered by the selective criteria of the available sources.’*

**NB: We did not include a specific section on Limitations because our comprehensive analysis is not affected by the constraints of working with second-hand sources, and the cognitive nature of our analysis allows us to consider the dataset as a whole despite the chronological span.**

b) The dataset section should include the sample size with exclusions (these are mentioned in the results section but should be described in the materials and methods section).

**The dataset we have considered comprises all published photos. Within the scope of our analysis, we excluded only three fragments given the low resolution of the published photographs. Accordingly, we have specified this in lines 350-356, as follows:**

*‘In this study, the dataset of the EOES is garnered from published materials [1-5] which provided only a selection of the fragments. We collected images for 112 EOES fragments from Diepkloof (93 made available out of 249), Klipdrift (17 out of 95), and Apollo 11 (2 out of 2) (S1 Table). Twelve are joint fragments composed of 2 to 11 specimens. Three fragments from Diepkfloof Rockshelter (D13, D68, and D73 in our dataset) were excluded from our statistical analysis, as they appear too worn out, and the image quality hinders a precise drawing of the motifs.*’

**4. Results and discussion.**
a) In my opinion, there are several statements that should be elaborated. For example, line 205 reads: “EOES were engraved with perceptually grounded basic geometric affordances, that is straight/curved lines, parallel/ secant segments, and right/non-right angles, and cognitively salient strategies”. I don’t fully grasp what is meant with ‘cognitively salient strategies’. Are the authors referring to visual saliency of low-level features, or to processing fluency? In general, the terminology should be better explained for readers who are not too familiar with cognitive science lingo.

**Thank you, we agree with the reviewer’s suggestion and we have improved the clarity throughout the entire manuscript. Regarding the sentence highlighted by the reviewer, we now specify that perceptual salience is evident in the selection of distinct geometric features. Please see lines 674-676:**

*‘The regular and repeated use of these properties suggests that the OES were engraved with perceptually salient geometric affordances, namely straight/curved lines, parallel/secant segments, and right/non-right angles.’*

**In addition, to further clarify which cognitive process we observe in the discussion we have modified the titles of two subsections in the ‘*Results and discussion*’ portion of the paper, as follows: ‘*Distribution of geometric features: the salience of parallelism and orthogonality*’ and ‘*Regularities in alignment strategies and angular patterns: toward a ‘geometric grammar’’.***

b) Line 291 claims that the patters exhibit a “cognitively traceable ‘grammar’”. This is an interesting conclusion but sadly it leads nowhere. For this to be supported, the authors should demonstrate if and how the production process follows a recurrent series of steps across the sample (e.g., certain types of lines often follow from others in a sequence), as it has been shown to be the case for stone-tool technologies (Stout et al., 2021). The mere repetition of segments and angles may show intentionality but does not constitute an action grammar. If I misunderstood, and this is actually what they found, it should be explained better and discussed in the framework of relevant literature.

**We agree with the reviewer, and we have significantly revised the discussion section (please see the section ‘Regularities in alignment strategies and angular patterns: toward a ‘geometric grammar’’) to explain our argument more clearly. We do not claim that an action grammar can be constituted by repetition of primitives, rather we emphasize that the hierarchical nesting of cognitive operations (iteration, rotation, translation, and embedding) reveals a structured and regulated system of composition. This system reflects what we have called a ‘geometric grammar’, that is a set of procedural rules through which the EOES patterns are traced, combined, and organized according to a stable internal logic. We have now revised the section as follows (please see lines 848-896):**

*‘This structural coherence of the dataset results from a series of operations applied to sequences of aligned parallel lines and repeated angular configurations, such as rotation (moving a feature around a fixed point by a certain number of degrees), translation (moving a feature to a given point in space maintaining its size and inclination), iteration (repeating features within the same hierarchical level), and embedding (nested features in additional hierarchical levels) (Fig 7). The engravings are created not just through the repetition of these features or operations, but through ruled-based procedures that can become superimposed, governing how features are related and assembled into complex patterns. For example, a set of iterated parallel lines can provide embedding for other sets of lines. Thus, combinatorial patterns can create an internal logic that moves in the direction of a foundational ‘geometric grammar’.*

*This internal geometric logic becomes especially evident in the hatched-band, grid, and diamond-shaped motifs, which show a deliberate construction of large, structurally coherent configurations through precise alignment and adherence to spatial organization. Hatched-band motifs, for instance, are created by first drawing two parallel lines that define a space within which another line, starting from the upper parallel and ending at the lower one, is iterated (Fig 7.1). This line, inclined up to 90° relative to the two parallel baselines, is then translated n times. The embedding of this second set of lines within the first results in hierarchically organized configuration. Interestingly, the different observed inclinations (acute* vs. *right angle) may imply distinct perceptual processing, as shape discrimination changes when rotation exceeds 40° [40].*

*Grid motifs show comparable operations: parallel segments are iterated to define a space within which other parallel segments, inclined 90° and starting from the upper parallel line, are embedded (Fig 7.2), as suggested by the upper-left portion of D19 in our dataset (S1 Table). Diamond-shaped motifs, by contrast, stand out because the space in which the configuration is embedded is not outlined by visible lines, but rather emerges as an abstract area made perceptible through the regularity of the composition itself, where iterative operations unfold (Fig. 7.3; K1, K2, K7, K10, K11 in S1 Table).’*

**Please note that, to clarify the operational procedures in making the patterns, we have added a new figure (Fig 7) illustrating the combinatorial and embedding processes. This figure was generated from a pseudo-code (plain-language algorithm) and now is included in the supplementary information (S2 Document).**

c) I miss a discussion on the internal variability of the sample. I mean, it would have been interesting to see whether and to what extent the fragments of each site differed from each other, perhaps alluding to different production strategies or local pattern preferences.

**We thank the reviewer for the comment. As shown in Figure 2, some chronological and site-specific preferences can be observed, and we have now explicitly acknowledged this, along with the reasons why we take the assemblage as a whole. Please see lines 307-341:**

*‘Accordingly, some site-specific and chronological preferences can be observed (Fig 2). To cite some examples, the diamond-shaped motif is only seen in Kilpdrift during the final HP phase, the orthogonal hatched band occurs exclusively in the intermediate and final phases in Diepkloof, and in Apollo 11 only the sub-parallel intersecting lines motif is present, while totally absent in Klipdrift.*

*However, we take the assemblage in its entirety, since the aim of this analysis is to study the cognitive underpinnings of the EOES: this approach allows us to capture overarching structural regularities. Methodologically, this choice is supported by the material, cultural, geographic, and chronological uniformity of the HP, which ensures that our comprehensive analysis is not hindered by the selective criteria of the available sources.’*

d) Line 349 starts discussing pattern variations and their possible causes (different tools, skill levels, etc.). Again, this is an interesting discussion that falls short. Elaborating how different tools and skills could have led to different patterns (supported ideally by experimental work) would offer a novel approach to these materials, in the way this has been done for figurative engravings (Rivero, 2016).

**We thank the reviewer for this observation that helped us recognize critical aspects of the subsection ‘*Variations from regular patterns and their salience*’. We agree that we have put too much emphasis on an aspect that, even though interesting, was not in the right place to be deepened. Therefore, we have changed the title of the subsection in *‘Spatial arrangements of intersection geometries: regular distances’* (line 903-904) and refined it to better highlight the spatial regularity of the EOES, in line with the cognitive focus. Lines 1035-1040 show that we have moderated the emphasis previously placed on this aspect:**

‘*These departures are perceptually salient as they deviate from the expected and recursive regularity [41,42]. The high degree of regularity observed in our analyses indicates technical skills with a precise execution of structured patterns (see for example larger rejoined fragments such as D49, D50, and D98, Fig 6), which suggests intentional variation. To further verify this possibility, specific analyses supported by experimental work and hands-on examination of the material are required and deserve a separate dedicated investigation*.’

**5. Conclusion**
The conclusion that this study offers a new way of understanding “the mechanisms by which geometric strategies were organized, transmitted, transformed, and stabilized within early human communities” (Line 369) seems to me an overstatement, given the gaps mentioned above. The following lines suggest that some of the questions raised in this review might be addressed in future work, so perhaps this analysis was premature and the authors should have waited until they could offer an more robust paper.

**The conclusion has been heavily re-written and we believe that now this issue has been addressed and corrected.**

**Cited references:
*** Henshilwood, C. S., van Niekerk, K. L., Wurz, S., Delagnes, A., Armitage, S. J., Rifkin, R. F., ... & Mienies, S. S. (2014). Klipdrift shelter, southern Cape, South Africa: preliminary report on the Howiesons Poort layers. Journal of Archaeological Science, 45, 284-303.
* Nel, T. H., & Haaland, M. M. (2023). Klipdrift Shelter, South Africa. In Handbook of Pleistocene Archaeology of Africa: Hominin behavior, geography, and chronology (pp. 1549-1561). Cham: Springer International Publishing.
* Rivero, O. (2016). Master and apprentice: evidence for learning in Palaeolithic portable art. Journal of Archaeological Science, 75, 89-100.
* Stout, D., & Chaminade, T. (2012). Stone tools, language and the brain in human evolution. Philosophical Transactions of the Royal Society B: Biological Sciences, 367(1585), 75-87.
* Texier, P. J., Porraz, G., Parkington, J., Rigaud, J. P., Poggenpoel, C., & Tribolo, C. (2013). The context, form and significance of the MSA engraved ostrich eggshell collection from Diepkloof Rock Shelter, Western Cape, South Africa. Journal of Archaeological Science, 40(9), 3412-3431.
* Texier, P. J., Porraz, G., Parkington, J., Rigaud, J. P., Poggenpoel, C., Miller, C., ... & Verna, C. (2010). A Howiesons Poort tradition of engraving ostrich eggshell containers dated to 60,000 years ago at Diepkloof Rock Shelter, South Africa. Proceedings of the National Academy of Sciences, 107(14), 6180-6185.
* Tylén, K., Fusaroli, R., Rojo, S., Heimann, K., Fay, N., Johannsen, N. N., ... & Lombard, M. (2020). The evolution of early symbolic behavior in Homo sapiens. Proceedings of the National Academy of Sciences, 117(9), 4578-4584.
* Vogelsang, R., Richter, J., Jacobs, Z., Eichhorn, B., Linseele, V., & Roberts, R. G. (2010). New excavations of Middle Stone Age deposits at Apollo 11 Rockshelter, Namibia: stratigraphy, archaeology, chronology and past environments. Journal of African Archaeology, 8(2), 185-218.
